# Targeting Myeloid FoxO1 Ameliorates Sepsis-induced Intestinal Injury by Modulating Tim4^+^ Macrophage Glycolysis

**DOI:** 10.7150/ijbs.119052

**Published:** 2026-01-01

**Authors:** Jiali Ni, Ruowen Zhang, Yaqi Pu, Yaoqi He, Wen Hu, Lengge Su, Yayi Hou, Huan Dou

**Affiliations:** 1 The State Key Laboratory of Pharmaceutical Biotechnology, Division of Immunology, Medical School, Nanjing University, Nanjing 210093, PR China.; 2 Jiangsu Key Laboratory of Molecular Medicine, Nanjing, 210093, PR China.; 3 Nanjing Drum Tower Hospital Clinical College of Nanjing University of Chinese Medicine, Nanjing 210093, PR China.

**Keywords:** FoxO1, Intestinal injury, Tim4, Macrophage, Glycolysis

## Abstract

Intestinal injury in sepsis contributes to life-threatening systemic infections, accompanied by disruptions in macrophage abundance and function. Forkhead Box O1 (FoxO1) is a critical transcription factor involved in regulating inflammatory responses; however, its role in sepsis-induced intestinal injury remains unclear. In this study, we found that FoxO1 expression was upregulated in intestinal macrophages of septic mice. To investigate its function, myeloid-specific FoxO1 conditional knockout (FoxO1^M-KO^) mice were established. Sepsis-induced intestinal barrier dysfunction and oxidative stress were significantly alleviated in these mice, along with improvements in systemic inflammation. Specifically, FoxO1 deletion increased the proportion of Tim4⁺ resident macrophages in intestinal lamina propria and Peyer's patches (PPs) of septic mice. Mechanistically, FoxO1 interacted with the corepressor Sin3a to restrict Tim4 transcription in macrophages. Functionally, FoxO1 knockdown reduced glycolysis in Tim4⁺ macrophages through MAP4K4 signaling, exerting an anti-inflammatory effect that mitigated intestinal injury. Adoptive transfer of Tim4-knockdown primary macrophages into septic mice reversed the protective effects observed in FoxO1^M-KO^ mice, underscoring the crucial role of FoxO1-regulated Tim4⁺ macrophages in inflammation. Furthermore, the traditional Chinese medicine Huashi Baidu formula (HSBD) inhibited FoxO1 in Tim4⁺ macrophages and ameliorated septic intestinal injury. In conclusion, this study reveals the immunomodulatory and inflammatory role of myeloid FoxO1, identifying it as a novel regulator and therapeutic target for septic intestinal injury.

## Introduction

Sepsis, defined as life-threatening organ dysfunction caused by a dysregulated immune response to infection, remains a major global health concern. In 2017, sepsis accounted for over 48.9 million cases and 11 million deaths worldwide, representing nearly 20% of global mortality 1, 2. In China, from 2017 to 2019, there were 806,728 sepsis-related deaths, contributing to a higher burden of sepsis hospitalizations than previously estimated 3. Sepsis-induced intestinal injury is frequently designated as the “center organ” or “engine” of multi-organ dysfunction 4-6. The gastrointestinal tract, a metabolically active and immunologically critical tissue, is particularly vulnerable to barrier disruption, microbial translocation, and endotoxin leakage, which exacerbate the systemic cytokine storm characteristic of sepsis 7-9. A retrospective cohort study found that acute gastrointestinal injury frequently occurred in patients with septic shock during the first week of intensive care unit (ICU) admission. Moreover, 12.2% of these patients developed acute gastrointestinal failure, which was associated with an increased risk of ICU mortality 10. Intestinal macrophages are widely distributed throughout the intestinal mucosal immune system, comprising gut-associated lymphoid tissues for gut immune surveillance, intestinal lamina propria and epithelium as effector sites 11-13. As indispensable parts of the innate immune system, intestinal macrophages act as gatekeepers of intestinal immune homeostasis, playing a crucial role in intestinal inflammation and mucosal repair. Depletion of macrophages using clodronate treatment further accentuated sepsis outcomes in mice, including increased mortality, worsened gut leakage, growth of fecal fungi and profound systemic inflammation 14. Enhancing the clearance function, anti-inflammatory cytokine secretion, and M2 polarization of intestinal macrophages has been shown to improve septic intestinal injury 15. Specific drugs, such as tetrahedral framework nucleic acids and naringin, have demonstrated protective effects against septic intestinal damage 16, 17. Therefore, regulating the localization, phenotype, and function of resident and recruited intestinal macrophages may represent an effective strategy to mitigate sepsis progression.

Forkhead Box O1 (FoxO1) is a critical transcription factor involved in cell proliferation, autophagy, metabolism and mitochondrial function across various diseases. Some studies have reported diverse roles of FoxO1 in septic organ injury, including downregulation of nuclear FoxO1 in cardiomyopathy, upregulation of FoxO1 in pulmonary endothelial injury, and decreased of FoxO1 acetylation in kidney injury 18-20. FoxO1 is abundantly expressed in myeloid cells, including macrophages 21. Inhibition of FoxO1 in macrophages has been shown to restore the pro-inflammatory responses caused by the deficiency of mechanistic target of rapamycin complex (mTORC) signaling 22. FoxO1 promoted the transcription of Toll-like receptor 4 (TLR4) in macrophages, enhancing inflammatory responses 23. Additionally, FoxO1 skewed macrophage polarization from the M2 phenotype toward the M1 phenotype, contributing to hepatic inflammation 21. However, the role of FoxO1 in macrophages during septic intestinal injury remains unknown.

In the present study, macrophage FoxO1 was identified as a powerful driver of septic intestinal injury by mediating the proportion and glycolysis of Tim4^+^ macrophages. Moreover, the traditional Chinese medicine Huashi Baidu formula (HSBD) effectively inhibited the expression of macrophage FoxO1, alleviating intestinal injury in septic mice, which is expected to be popularized and studied in the future. Our study demonstrate that FoxO1 emerged as a novel therapeutic target for septic intestinal injury, with druggable potential supported by its central role in macrophage- mediated inflammation.

## Material and Methods

### Mice

Male C57BL/6 mice aged 8-10 weeks were purchased from Cavens Experimental Animal Co., Ltd. (Changzhou, Guangdong, China) and maintained under specific pathogen-free (SPF) conditions at a light-dark cycle (12:12 h light-dark cycle). CRISPR/Cas9 technology was used to modify FoxO1 gene. The predicted promoter region and exon1 of FoxO1-201 (ENSMUST00000053764.6) transcript is recommended as the knockout region. Myeloid FoxO1 knock out mice were generated by crossing C57BL/6JGpt-FoxO1^Cas9-KO^ with C57BL/6JGpt-Lyz2^em1Cin(iCre)^/Gpt mice (GemPharmatech Co., Ltd., Nanjing, Jiangsu, China), resulting in the loss of function of FoxO1 in myeloid cells through myeloid-specific lysozyme 2 (Lyz2) promoter. Mice were acclimatized in housing conditions for at least one week. Mice experiments were approved by the Institutional Animal Care and Use Committee, Affiliated Drum Tower Hospital, Medical School of Nanjing University (IACUC assurance number: 2023AE01036).

### Murine sepsis model

Cecal ligation and puncture (CLP) mice model was constructed as previously described. We used 4-0 silk suture to ligate approximately 40% cecum for the induction of mild-grade sepsis 24, 60% cecum for the induction of high-grade sepsis in the survival monitoring experiments 25. Immediately after surgery, administer subcutaneous injections of prewarmed saline to the mice (1 mL per mice). Sham-operated group underwent a similar procedure without CLP. The survival rate of mice was monitored 7 days after the surgery, health and behavior of mice were observed every day to check for the living state. In accordance with the published guidelines, observe mice to determine humane endpoints 26.

Huashi Baidu formula (HSBD) is approved by the National Medical Products Administration (NMPA). It was purchased from Guangdong Efong Biopharmaceutical Co. Ltd., China. HSBD formula was dissolved in heated sterile distilled water to achieve a storage concentration of 0.25 g/mL. Based on previous study, we have determined three dosage concentrations ranging from low to high 27. CLP septic mice were randomized to receive a i.g. dose (low: 0.7 g/kg; medium: 1.4 g/kg; high: 2.8 g/kg) of HSBD, administered every 8h after the surgery, respectively. Dexamethasone (DXMS) tablets were purchased from Guangdong South Land Pharmaceutical Co. Ltd., and dissolved in sterile distilled water at 0.5 mg/mL, and CLP septic mice were given 2.0 mg/kg DXMS by gavage every 8 hours after the surgery. The sham group was given proper volume of sterile distilled water. At 24h post-CLP, mice were eventually euthanized by asphyxiation via carbon dioxide.

Lipopolysaccharide (LPS)-induced sepsis model was constructed by intraperitoneal injection of 10 mg/kg LPS (Sigma-Aldrich, St. Louis, MO, USA). After 24 h, mice were eventually euthanized by asphyxiation via carbon dioxide. To evaluate the survival of mice, after a subsequent lethal dose LPS (35 mg/kg) or dispensing liquid injection, mice were monitored regularly, and survival was recorded for 7 days.

### Lung wet/dry weight ratio

The upper lobe of the right lung was excised and weighted (wet weight), then the lung tissues were placed in an incubator at 60°C for 72 h to obtain dry weight (dry weight). The wet/dry weight ratio was calculated.

### Adoptive transfer

1 mL 6% starch broth medium were intraperitoneally injected into C57BL/6 mice. Two days later, mice were euthanized to collect peritoneal macrophages 28. Sacrificed mice were disinfected in 75% ethanol for 5-10 min, and then were fixed on the dissection table. We used scissors and tweezers to carefully cut the abdomen skin layer and completely separate the skin layer from the muscular layer, and used a syringe to aspirate peritoneal fluid. Aseptic operations should be maintained at all times. Peritoneal fluid was centrifuged at 300 g for 5 min, and resuspended in Ammonium-Chloride-Potassium (ACK) Lysing Buffer for 2 min to remove red blood cells. After termination of lysis with phosphate buffered saline (PBS) and centrifugation washing, peritoneal cell precipitation was resuspended in DMEM with 10% FBS and 1% penicillin-streptomycin. Peritoneal cells were cultured for 2h, through cell adherent to purify peritoneal macrophages.

Mouse Tim4-siRNA (NM_178759.4) and negative control (NC) mimics were procured from RiboBio Co., Ltd. (Guangzhou, Guangdong, China) (Tim4-siRNA mimic: 5'-CAAGAATCATCTCCAGGAA-3'). RFect small nucleic acid transfection reagent was used for transfecting 72h (RiboBio). The knock-down Tim4 macrophages and NC macrophages were harvested with pancreatin (Gibco, Grand Island, NY, USA) and lidocaine (Mackin, Shanghai, China) and injected i.p. into mice at the day before they underwent CLP (1.5×10^6^ cells per mice) 29.

### Cell lines and cell culture

The murine macrophage cell line RAW264.7 cells was purchased from Cell Bank of Chinese Academy of Sciences, and maintained in DMEM supplemented with 10% FBS at 37 °C in a 5% CO₂ atmosphere. To simulate the inflammatory environment *in vitro*, we used LPS (1 μg/mL) to stimulate cells for 24 h.

### Isolation of Tim4^+^ macrophages

Peritoneal cells were obtained as the above protocol. We isolated Tim4^+^ primary peritoneal macrophages by staining peritoneal cells with anti-Tim4 antibody (clone RMT4-54)-APC (BioLegend, San Diego, CA, USA) and subsequently isolating the labeled cells with anti-APC microbeads (Miltenyi Biotec, Bergisch Gladbach, Germany).

### Cell transfection

Mouse FoxO1 (NM_019739.3) was cloned into the recombinant pcDNA3.1 eukaryotic expression vector (Generay, Shanghai, China), mouse Sin3a (NM_001110350.2) and its mutants (△930-Sin3a, △536-539-Sin3a, △565-567-Sin3a, △536-567-Sin3a) were cloned into the recombinant pEGFP-N1 eukaryotic expression vector (Generay). All plasmids were verified by DNA sequencing. Overexpressed plasmids and empty plasmids were transfected into cells with RFect plasmid DNA transfection reagent for 48 h (BIOG, Changzhou, Guangdong, China).

Negative control (NC) and Sin3a-siRNA and FoxO1-siRNA mimics were procured from RiboBio. RFect small nucleic acid transfection reagent was used for transfection (FoxO1-siRNA mimic: 5'- GGGAGAATGTTCGCTTTCT-3', Sin3a-siRNA mimic: 5'- CAGAGCAGCTTATGTCAGA -3').

### Intestinal barrier permeability analysis

After 24h of CLP modeling, mice were gavaged with FITC-Dextran (25 mg/mL) (Cat. NO.: HY-128868, MedChem Express, Monmouth Junction, NJ, USA) at a volume of 20 μl/g. Serum samples were collected four hours later, and serum was diluted four-fold. A standard curve was prepared by serial dilution of FITC-Dextran in PBS. The fluorescence intensity in both samples and standard samples was measured by using a multifunctional microwell detector (BioTek, Winooski, VT, USA) to read at an excitation of 485 nm and an emission of 528 nm. The concentration of FITC-Dextran in each sample was determined by comparing the fluorescence intensity with the known concentrations of the standard curve.

### Measurement of superoxide dismutase (SOD), malondialdehyde (MDA), nitric oxide (NO)

Small intestine tissues were homogenized at 4 °C, and were centrifuged at 12,000 g at 4 °C for 5 min. The supernatants were collected for SOD, MDA and NO assay. The enzyme activity of SOD was detected by SOD assay kit (Cat. NO.: S0101S, Beyotime, Shanghai, China) according to the manufacturer's instructions. The level of MDA and NO was detected by MDA assay kit (Cat. NO.: S0131S, Beyotime) and NO assay kit (Cat. NO.: S0021S, Beyotime) according to the manufacturer's instructions.

### Flow cytometry

We used the previous protocol to isolate immune cells in small intestinal lamina propria and PPs 30, 31. Single-cell suspension was prepared and stained according to different staining schemes. The sample tube was blocked with Fc Blocker (101320, Biolegend) for 10 min. 7-AAD (Fcmas, Nanjing, Jiangsu, China), anti-CD45 antibody (clone 30-F11)-Percp (Biolegend), anti-CD11c antibody (clone N418)-Alexa Flour 647 (Biolegend), anti-CD45R antibody (clone RA3-6B2)-FITC (eBioscience, San Diego, CA, USA), anti-CD4 antibody (clone RM4-5)-BB700 (BD Biosciences, San Jose, CA, USA), anti-CD317(BST2) antibody (clone 927)-Alexa Fluor 700 (Biolegend), anti-MHCII antibody (clone AF6-120.1)-PE (eBioscience), anti-CX3CR1 antibody (clone SA011F11)-PE/Dazzle 594 (Biolegend), anti-Tim4 antibody (clone RMT4-54)-PE/Cyanine7 (Biolegend) were used to stain Tim4^+^ macrophages in PPs. 7-AAD, anti-CD45 antibody (clone 30-F11)-PerCP (Biolegend), anti-CD11b antibody (clone M1/70)-PE/Cy7 (Biolegend), anti-CD64 antibody (clone X54-5/7.1)-PE (Biolegend), anti-Ly6C antibody (clone HK1.4)-PE/Dazzle594 (Biolegend), anti-MHCII antibody (clone M5/114.15.2)-FITC (Biolegend), anti-Tim4 antibody (clone RMT4-54)-APC (Biolegend), anti-CD4 antibody-BB700 were used to stain Tim4^+^ macrophages in the lamina propria of the small intestine. After 30 min of dark staining, cells were washed twice with PBS, and detected with flow cytometer (CytoFLEX S, Beckman, Miami, FL, USA).

To further detected macrophage FoxO1 in small intestine, we used the following new antibody panel to stain surface markers for the identification of macrophages: A. PPs macrophages: Zombie NIR (Biolegend), anti-CD45 antibody-Percp (Biolegend), anti-CD11c antibody-Alexa Flour 647 (Biolegend), anti-CD45R antibody-PE (eBioscience), anti-CD4 antibody-BB700 (BD Biosciences), anti-CD317 (BST2) antibody-Alexa Fluor 700 (Biolegend), anti-MHCII antibody-APC/Cy7 (eBioscience), anti-CX3CR1 antibody-PE/Dazzle 594 (Biolegend), anti-Tim4 antibody-PE/Cyanine7 (Biolegend); B. Macrophages in the lamina propria of the small intestine: Ghost Dye™ Violet 510 (Tonbo, San Diego, CA, USA), anti-CD45 antibody-PerCP Cy5.5 (Biolegend), anti-CD11b antibody-PE/Cy7 (Biolegend), anti-CD64 antibody-PE (Biolegend), anti-Ly6C antibody-PE/Dazzle594 (Biolegend), anti-MHCII antibody-APC/Cy7 (Biolegend), anti-Tim4 antibody-APC (Biolegend), anti-CD4 antibody-BV650. Following surface staining, cells were fixed and permeabilized using the True-Nuclear™ Transcription Factor Buffer Set (Biolegend), according to the manufacturer's instructions. Cells were then incubated intracellularly with an anti-FoxO1 antibody (clone C29H4)- Alexa Flour 488 for 30 minutes in the dark. Data were acquired on a flow cytometer (CytoFLEX).

### Dual luciferase assay

JASPAR (https://jaspar.elixir.no/) database was used to predicate the binding site between FoxO1 and Tim4 promoter. The FoxO1 binding Tim4 promoter fragment was cloned into the pGL3 luciferase vector to generate a wild-type (WT) reporter plasmid. The top three binding sequences with the highest scores were selected and mutated (TTTGTTTTCC was replaced by TTGCAGCGCC, ACAGTTTACAC was replaced by ACAACGGCTAC, CATGTGTACTC was replaced by CATTCACGATC) to obtain mutant plasmids (MUT). RAW264.7 cells were co-transfected with the FoxO1 (or empty) plasmid, a Renilla luciferase (Rluc) expression vector CV045-TK (GeneChem, Shanghai, China), and either the WT or MUT luciferase plasmids. It was measured using a dual luciferase assay system (Vazyme, Nanjing, Jiangsu, China) and then normalized to Rluc activity.

### Co-immunoprecipitation (Co-IP)

Co-immunoprecipitation was performed as previously described 32. Protein was extracted from RAW264.7 cells under LPS treatment. We used anti-FoxO1 antibody or IgG antibody to combine FoxO1 in protein, and used Protein A/G agarose beads (Bioworld, Nanjing, Jiangsu, China) to precipitate complex. The products were washed with lysis buffer and prepared for western blot. Protein in RAW264.7 cells transfected Sin3a and mutant plasmids was precipitated by anti-GFP antibody (Bioworld), the remaining steps were the same as above.

### Chromatin immunoprecipitation (ChIP) assay

The ChIP assay was conducted using a ChIP-IT Express Enzymatic Kit (Active Motif, Carlsbad, CA, USA). Following formaldehyde cross-linking, nuclei were isolated and lysed. The chromatin was sheared enzymatically to obtain 100-200 bp fragments, which were then immunoprecipitated with an anti-FoxO1 antibody (Cell Signaling Technology, Danvers, MA, USA) or control IgG (CST) coupled to protein G agarose. The immunoprecipitated DNA was then washed, eluted, and purified for PCR analysis. The binding sites of FoxO1 and Tim4 promoter was predicted by the JASPAR database and further verified by ChIP-PCR (Forward primer: GTGATTGATTGCATCCTGC; Reverse primer: CCTGAGTGTGCCTGAGCATCTG).

### Oxygen consumption rate (OCR) and extracellular acidification rate (ECAR)

To measure ECAR and OCR in real-time, primary Tim4^+^ macrophages were isolated from mice, seeded at 2×10^5^ cells/well in XFe96 cell culture microplates. RAW264.7 cells were seeded at the density of 5×10^4^ cells per well in XFe96 cell culture microplates. One hour prior to reading cells were washed twice with, and then cultured in Seahorse XF base medium (Agilent Technologies) supplemented with 1 mM pyruvate, 2 mM L-glutamine and 10 mM glucose in an incubator without CO_2_. OCR of Tim4^+^ primary macrophages were measured under basal conditions prior to sequential treatment of cells with electron train chain inhibitors 1.5 μM oligomycin, 2 μM FCCP, and 0.5 μM antimycin A and rotenone (Seahorse XF Cell Mito Stress Test kit, Agilent, Santa Clara, CA, USA). OCR of RAW264.7 cells were measured under basal conditions prior to sequential treatment of cells with electron train chain inhibitors 1.5 μM oligomycin, 1 μM FCCP, and 0.5 μM antimycin A and rotenone (Seahorse XF Cell Mito Stress Test kit, Agilent). ECAR were measured under basal conditions prior to sequential treatment of cells with 0.5 μM antimycin A and rotenone and 50 mM 2-DG (Seahorse XF Glycolytic Rate Assay Kit, Agilent). The data was detected by Seahorse XFe96 analyzer (Agilent).

### Enzyme-linked immunosorbent assay (ELISA)

Blood from mice was collected and supernatant was obtained after centrifuging at 12,000 rpm for 15 min. Sera Interleukin-6 (IL-6) was analyzed using a Mouse IL-6 Kit (Beijing 4A Biotech Co., Ltd, Beijing, China) and the sera was applied at a dilution of 1:1 according to manufacturer's instructions. Sera TNF-α was analyzed using a Mouse TNF-α Kit (Invitrogen, Carlsbad, CA, USA) and the sera was applied at a dilution of 1:2 according to manufacturer's instructions.

### Glucose uptake, lactate production assays

According to the manufacturer's instructions, cells were lysed by sonication, the Maltose and Glucose Assay Kit (Solarbio, Beijing, China) was used to detect the glucose concentration in RAW264.7 cells and primary Tim4^+^ macrophages, while the L-Lactate Assay Kit (Solarbio, Beijing, China) was used to determine the concentration of lactic acid in RAW264.7 cells and primary Tim4^+^ macrophages.

### RNA extraction, sequencing and analysis

After we collected the single-cell suspension of PPs as the above protocol, cells were cultured for 2h, through cell adherent to purify PPs macrophages. We extracted PPs macrophages RNA using TRIzol reagent (Vazyme) from sham or CLP mice for RNA sequencing. Raw sequencing reads were processed by removing adapter sequences and low-quality reads to generate clean data. The clean reads were aligned to reference genome using the HISAT2. Gene counts were obtained with HTSeq, and gene expression levels were quantified using FPKM. Differentially expressed genes (DEGs) were identified with the DESeq2 algorithm, using a significance threshold of |Fold Change|>2 and an adjusted P value<0.05. We then integrated the related matrix with the LM22 characteristic gene matrix using R code from the CIBERSORT to analyze the immune infiltration.

### Molecular docking

AlphaFold2 was used to predict the crystal structures of FoxO1 and Sin3a proteins. These predicted structures were then prepared with the Protein Preparation Wizard module in Schrodinger software, which involved steps including protein pretreatment, native ligand regeneration, hydrogen bond assignment optimization, and energy minimization. Subsequently, protein-protein docking was performed, and the conformation with the lowest binding energy was selected from the search results. The protein-ligand interactions within the final complex, such as hydrogen bonds and their bond lengths, were analyzed using PyMol.

### Histological analysis

The small intestine, liver and lung tissues of mice was isolated and completely soaked in 4% Paraformaldehyde (PFA) for fixation. Hematoxylin/eosin (HE) staining was used to evaluate the morphological changes of tissues. Sections were randomly selected and photographed. The histological score of lung tissues was scored on the basis of the following categories from 0 to 4 (absent to severe): lung alveolar congestion, hemorrhage, neutrophil or leukocyte infiltration, and thickness of the alveolar wall 33. The histological score of the small intestine was scored on the basis of a 0-4 grading scale as previously reported 34. The histological score of the liver was also scored on the basis of a 0-4 grading scale, including three parameters (inflammation, necrosis/abscess formation, and thrombus formation) 15.

### RNA extraction and quantitative real-time PCR (qPCR)

We used TRIzol reagent (Vazyme) to extract total RNA from cells or tissues. Subsequently, the extracted RNA was reverse-transcribed into cDNA using HiScript ® II QRT SuperMix kit (Vazyme). The resultant cDNA was then subjected to qPCR assay using SYBR qPCR Master Mix (Vazyme). Gene expression levels were normalized to GAPDH or ACTB and relative quantitation was determined using the 2^-ΔΔCt^ method. The corresponding primer sequences are provided in Table S1.

### Western blot analysis

Tissues and cells lysis were achieved using RIPA buffer (P0013B, Beyotime) containing phosphatase inhibitor cocktail (GRF102, EpiZyme, Shanghai, China) and proteinase inhibitor cocktail (GRF101, EpiZyme). Proteins from the lysates were separated via SDS-PAGE gel and electrotransferred, the detailed procedure was performed as previously described 35. The primary and secondary antibodies were diluted in accordance with the recommended concentration range of the instructions, and incubated overnight in a shaking table at 4°C. The primary antibodies included: anti-ZO-1 antibody (21773-1-AP, Proteintech, Rosemont, IL, USA) at a 1:1000 dilution, anti-Occludin antibody (13409-1-AP, Proteintech) at a 1:1000 dilution, anti-β-actin antibody (BS6007M, Bioworld) at a 1:4000 dilution, anti-FoxO1 antibody (2880S, CST, USA) at a 1:1000 dilution, anti-Tim4 antibody (sc-390805, Santa Cruz Biotechnology, Santa Cruz, CA, USA) at a 1:1000 dilution, anti-GLUT1 antibody (R380464, ZENBIO, Chengdu, Sichuan, China) at a 1:1000 dilution, anti-PKM2 antibody (15822-1-AP, Proteintech) at a 1:1000 dilution, anti-MCT1 antibody (20139-1-AP, Proteintech) at a 1:2000 dilution, anti-HK2 antibody (22029-1-AP, Proteintech) at a 1:5000 dilution, anti-LDHA antibody (R24822, ZENBIO) at a 1:1000 dilution, anti-GAPDH antibody (10494-1-AP, Proteintech) at a 1:5000 dilution, and the secondary antibodies included: HRP* Goat Anti-Rabbit IgG(H+L) (RS0002, Immunoway, Plano, TX, USA), HRP* Goat Anti-Mouse IgG(H+L) (FMS-MS01, Fcmacs). Finally, minichemTM chemiluminescence imaging system (Sagecreation, Beijing, China) was used to capture images and analyzed gray values by LANE 1D Analysis software.

### Statistical analysis

All data were showed as mean±standard deviation (means±SD) and analyzed by GraphPad Prism8.0 software (GraphPad Software Inc., San Diego, CA, USA). All data presented are representative of at least three independent experiments. Statistical comparisons between two groups were performed with the Student's t-test, and among multiple groups by one-way analysis of variance (ANOVA), with P<0.05 considered significant.

## Results

### FoxO1 is upregulated in the intestinal macrophages of septic mice

**Figure 1:**
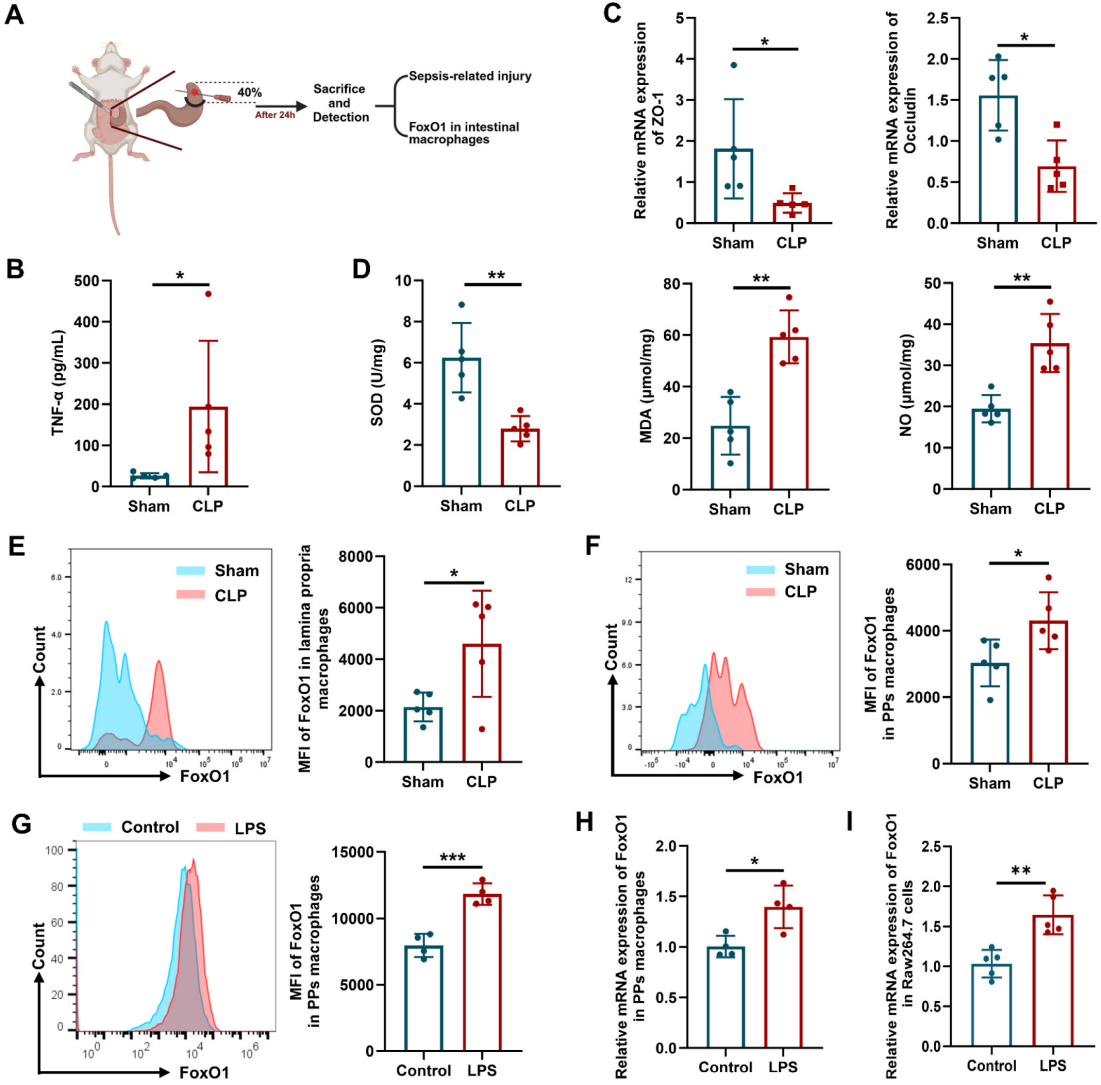
** FoxO1 is upregulated in the intestinal macrophages of septic mice. (A)** Scheme of experimental. **(B)** The level of TNF-α in serum from sham and CLP mice detected by ELISA (n=5). **(C)** Relative mRNA expression of ZO-1 and Occludin in small intestine tissues from sham and CLP mice detected by qPCR (n=5). **(D)** The enzyme activity of SOD, the level of MDA and NO in small intestine tissues from sham and CLP mice (n=5). **(E)** Mean fluorescence intensity (MFI) of FoxO1 in macrophages from lamina propria in sham and CLP mice detected by flow cytometry, showed as representative histogram (n=5). **(F)** MFI of FoxO1 in macrophages from PPs in sham and CLP mice detected by flow cytometry, showed as representative histogram (n=5). **(G)** MFI of FoxO1 in primary macrophages from PPs under LPS treatment (1 μg/mL) for 24 h detected by flow cytometry (n=4). **(H)** Relative mRNA expression of FoxO1 in primary macrophages from PPs under LPS treatment (1 μg/mL) for 24 h detected by qPCR (n=4). **(I)** Relative mRNA expression of FoxO1 in Raw264.7 cells under LPS treatment (1 μg/mL) for 24 h by qPCR (n=5). Data represent the mean scores±SD. *P<0.05, **P<0.01.

Intestinal injury is a hallmark of CLP-induced sepsis, characterized by barrier disruption and impaired antioxidant status 36. In the CLP mouse model (Fig. 1A), levels of the pro-inflammatory cytokine tumor necrosis factor-α (TNF-α) in peripheral blood were elevated, whereas the expression of the tight junction proteins (ZO-1 and Occludin) in the intestinal epithelium was reduced (Fig. 1B, C). As shown in Fig 1D, superoxide dismutase (SOD) activity in the small intestine was decreased, while levels of malondialdehyde (MDA) and nitric oxide (NO) were increased, indicating enhanced oxidative stress and inflammation in the intestine. Intestinal macrophages are distributed across multiple gut layers and exhibit considerable heterogeneity. Therefore, we focused on the macrophages in the intestinal lamina propria (identified as CD45^+^CD11b^+^CD64^+^Ly6C^-^MHCII^+^) and Peyer's patches (PPs, which belong to GALT, identified as CD45^+^CD4^+^B220^-^CX3CR1^+^BST2^+^CD11c^+^MHCII^low^) using flow cytometry 37, 38. The gating strategies for intestinal macrophages were shown in Fig. S1. The mean fluorescence intensity (MFI) of FoxO1 in these macrophages was significantly elevated in CLP mice (Fig. 1E, F). To further investigate, we enriched primary macrophages from PPs of mice, and stimulated them with LPS. LPS treatment markedly increased the mRNA and protein expression of FoxO1 in PPs macrophages (Fig. 1G, H). Similarly, FoxO1 expression was upregulated in RAW264.7 cells following LPS exposure (Fig. 1I). These findings suggest that FoxO1 in intestinal macrophages may contribute to intestinal damage during sepsis.

### Intestinal injury is relieved in FoxO1^M-KO^ septic mice

**Figure 2:**
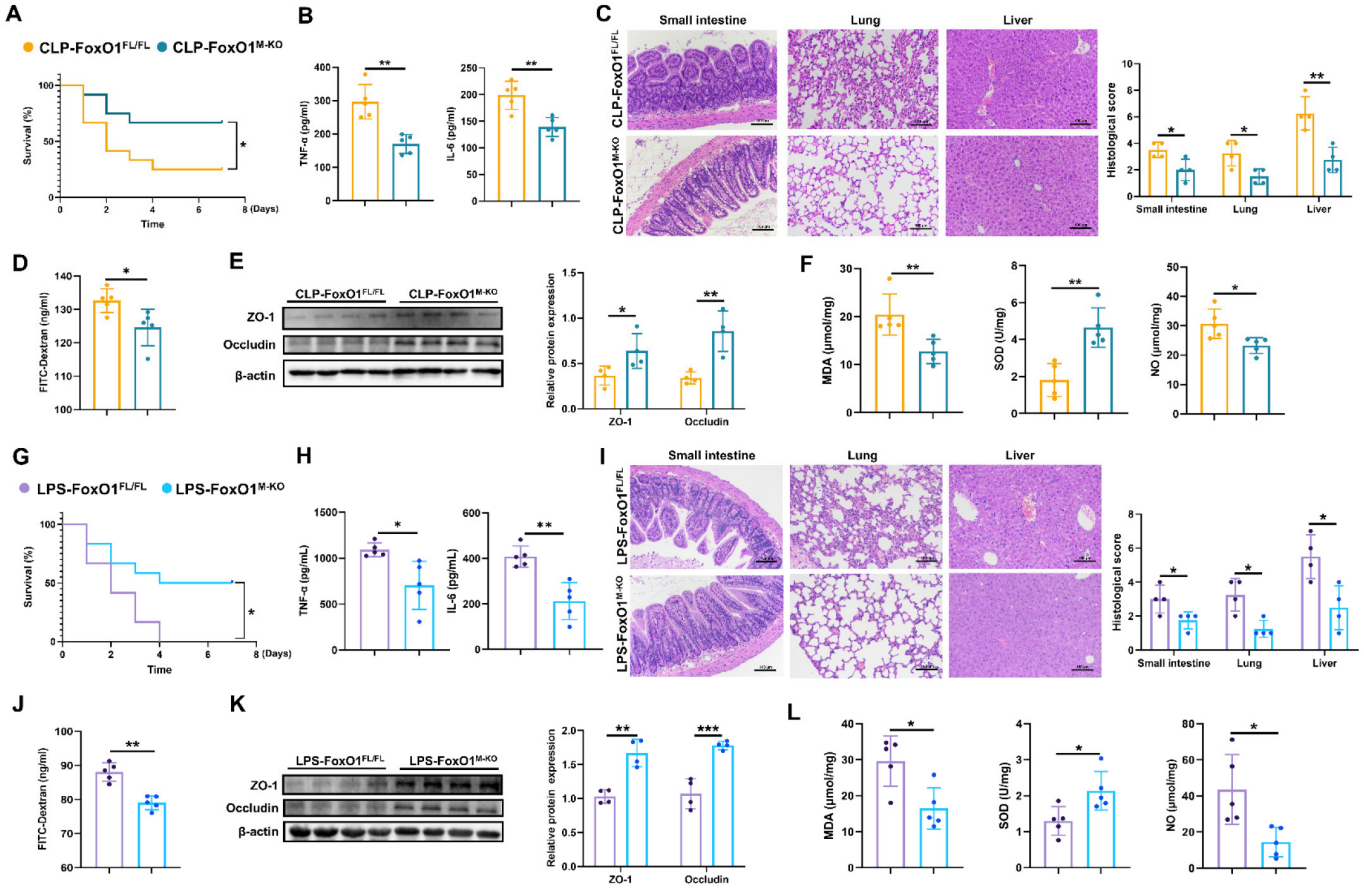
** Intestinal injury is relieved in FoxO1^M-KO^ septic mice. (A)** Survival curve in FoxO1^FL/FL^ and FoxO1^M-KO^ CLP mice with 7 days (n=12). **(B)** The level of TNF-α and IL-6 in serum from FoxO1^FL/FL^ and FoxO1^M-KO^ CLP mice determined by ELISA (n=5). **(C)** Small intestine, lung and liver histopathological evaluation by HE staining. Representative images of these tissues from FoxO1^FL/FL^ and FoxO1^M-KO^ CLP mice (above). Statistical results of histological score in tissues (below) (n=4). **(D)** The fluorescence of FITC-Dextran in serum from FoxO1^FL/FL^ and FoxO1^M-KO^ CLP mice (n=5). **(E)** Relative protein expression of ZO-1 and Occludin in small intestine tissues from FoxO1^FL/FL^ and FoxO1^M-KO^ CLP mice detected by WB, the statistical results were showed as histograms (n=4). **(F)** The enzyme activity of SOD, the level of MDA and NO in small intestine tissues from FoxO1^FL/FL^ and FoxO1^M-KO^ CLP mice (n=5). **(G)** Survival curve in FoxO1^FL/FL^ and FoxO1^M-KO^ LPS-induced septic mice with 7 days (n=12). **(H)** The level of TNF-α and IL-6 in serum from FoxO1^FL/FL^ and FoxO1^M-KO^ LPS-induced septic mice determined by ELISA (n=5). **(I)** Small intestine, lung and liver histopathological evaluation by HE staining. Representative images of these tissues from FoxO1^FL/FL^ and FoxO1^M-KO^ LPS-induced septic mice (above). Statistical results of histological score in tissues (below) (n=4). **(J)** The fluorescence of FITC-Dextran in serum from FoxO1^FL/FL^ and FoxO1^M-KO^ LPS-induced septic mice (n=5). **(K)** Relative protein expression of ZO-1 and Occludin in small intestine tissues from FoxO1^FL/FL^ and FoxO1^M-KO^ LPS-induced septic mice detected by WB, the statistical results were showed as histograms (n=4). **(L)** The enzyme activity of SOD, the level of MDA and NO in small intestine tissues from FoxO1^FL/FL^ and FoxO1^M-KO^ LPS-induced septic mice (n=5). Data represent the mean scores±SD. *P<0.05, **P<0.01, ***P<0.001.

To evaluate the role of macrophage FoxO1 in septic intestinal injury, we generated FoxO1^M-KO^ mice and induced septic model. Following validation of knockout efficiency (Fig. S2A, B), CLP surgery was performed. Myeloid-specific FoxO1 conditional knockout (FoxO1^M-KO^) mice exhibited a significantly higher survival rate than FoxO1^FL/FL^ mice (Fig. 2A). The production of proinflammatory cytokines, including TNF-α and interleukin-6 (IL-6), in peripheral blood was markedly reduced in FoxO1^M-KO^ mice (Fig. 2B). Histological analysis revealed substantial improvements in multiple organs of FoxO1^M-KO^ mice, including reduced pulmonary erythema, inflammatory cell infiltration, and edema, as well as decreased hepatic central vein congestion and inflammatory infiltration (Fig. 2C). In the intestine, FoxO1 deletion alleviated villous distortion, reduced blunting, and diminished inflammatory cell infiltration in the mucosa (Fig. 2C). To evaluate intestinal barrier integrity, FITC-dextran was orally administered, and serum fluorescence was measured. FoxO1^M-KO^ mice exhibited lower fluorescence intensity, indicating improved intestinal barrier function (Fig. 2D). Additionally, the expression of ZO-1 and Occludin in intestinal epithelial tissues were higher in FoxO1^M-KO^ mice (Fig. 2E). In the small intestine of FoxO1^M-KO^ mice, SOD activity was elevated, whereas MDA and NO levels were reduced, indicating decreased oxidative stress (Fig. 2F). The results demonstrated that myeloid FoxO1 knock out protected the small intestine tissues from oxidative stress. Consistent with these observations, in LPS-induced septic models, FoxO1^M-KO^ mice exhibited improved survival, reduced TNF-α and IL-6 levels in peripheral blood (Fig. 2G, H), attenuated tissue inflammation (Fig. 2I), and mitigated intestinal barrier damage and oxidative stress (Fig. 2J-L). To determine whether FoxO1 deletion affects intestinal homeostasis under normal conditions, we examined intestinal integrity in FoxO1^M-KO^ mice and littermate controls without septic challenge. There were no significant differences in intestinal barrier function and oxidative stress between the two groups (Fig. S2C-E), highlighting the specific role of myeloid FoxO1 during acute inflammatory responses.

### Myeloid FoxO1 deficiency enhances the anti-septic intestinal injury of Tim4^+^ macrophages

**Figure 3:**
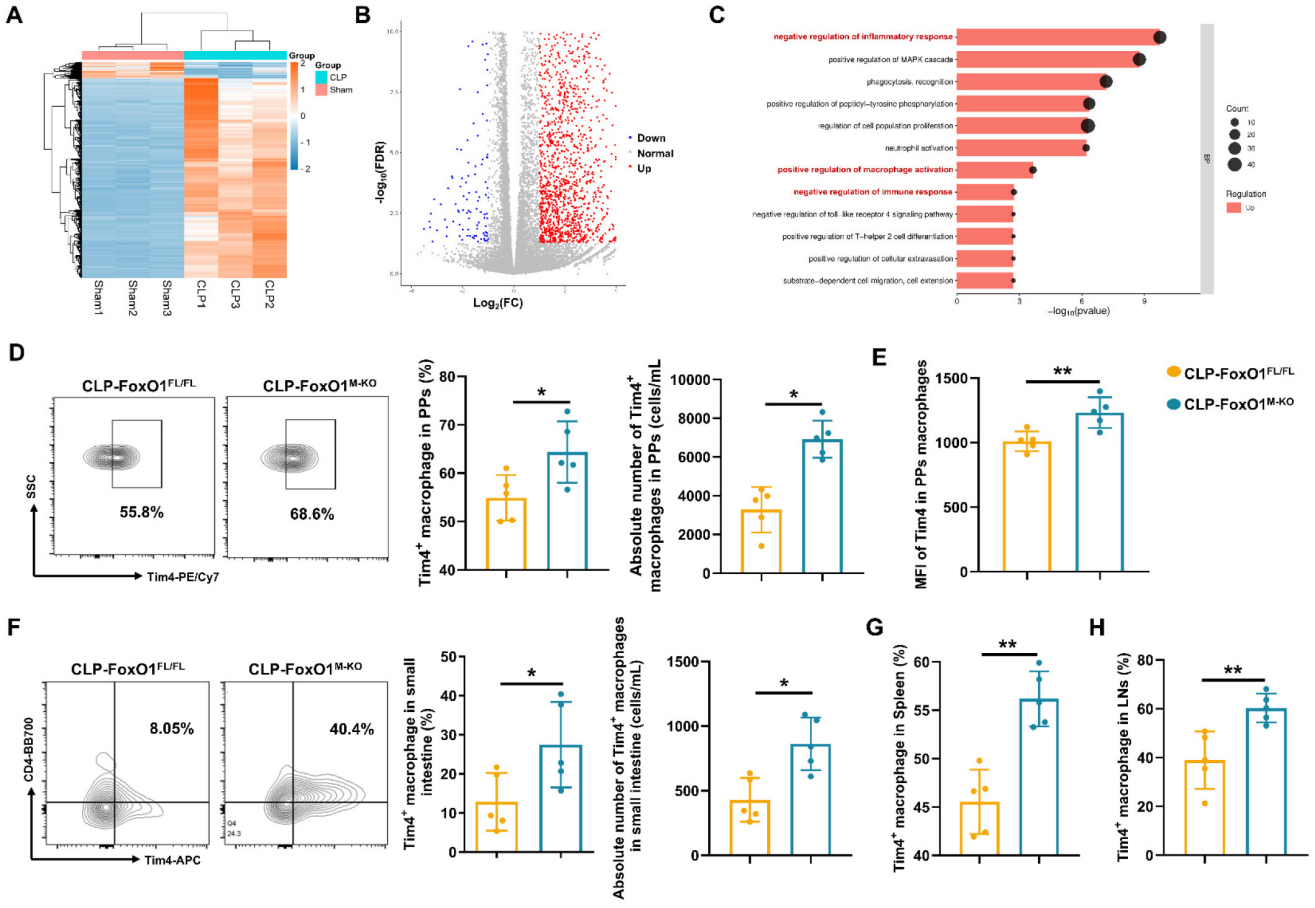
** Myeloid FoxO1 deficiency enhances the anti-septic intestinal injury of Tim4^+^ macrophages. (A)** Expression levels of differentially expressed genes (DEGs) in PPs macrophages from sham and CLP mice were shown as heatmap. **(B)** Volcano plot of the DEGs between sham and CLP group. Each point represents the average value of one transcript, which red means up-regulated, blue means down-regulated and grey means no difference. **(C)** GO analysis of biological process. **(D)** The frequency and absolute number of Tim4^+^ macrophages in PPs were determined by flow cytometry. Representative of flow cytometry scatter plot from each group (n=5). **(E)** MFI of Tim4 in PPs macrophages (n=5). **(F)** The frequency and absolute number of Tim4^+^ macrophages in small intestine were determined by flow cytometry. Representative of flow cytometry scatter plot from each group (n=5). **(G)** The proportion of Tim4^+^ macrophages in spleen were determined by flow cytometry (n=5). **(H)** The proportion of Tim4^+^ macrophages in inguinal lymph nodes were determined by flow cytometry (n=5). Data represent the mean scores±SD. *P<0.05, **P<0.01.

To investigate the effects of myeloid FoxO1 knockout on intestinal macrophages under septic conditions, we isolated PPs macrophages from sham and CLP mice for transcriptomic analysis. A total of 2,117 genes were upregulated, and 175 genes were downregulated (Fig. 3A, B). Gene Ontology (GO) analysis indicated prominent enrichment in pathways related to the “negative regulation of inflammatory response”, “positive regulation of macrophage activation”, and “negative regulation of immune response” (Fig. 3C). Thus, it indicated that intestinal macrophages in FoxO1^M-KO^ septic mice might exhibit a transition towards an anti-inflammatory and tissue-repairing phenotype. T cell immunoglobulin and mucin domain containing 4 (Tim4) is a phosphatidylserine receptor expressed on macrophages, and has been described as a specific marker for labelling tissue-resident macrophages in the intestine 39, 40. Previous study already found that Tim4^+^ subset in the intestinal macrophages showed heterogeneous expression compared with Tim4^-^ subset by transcriptional profiling, which indicated the anti-inflammatory functions of Tim4^+^ macrophages in the gut 39, 41. Using defined gating strategies for Tim4⁺ intestinal macrophages (Fig. S1), we found that myeloid FoxO1 knockout contributed the accumulation of Tim4^+^ macrophages and the increased MFI of Tim4 in PPs macrophages from CLP mice (Fig. 3D, E). The proportion and absolute number of Tim4^+^CD4^+^ macrophages from lamina propria were also increased in FoxO1^M-KO^ CLP mice (Fig. 3F). In LPS-induced septic models, FoxO1^M-KO^ mice also exhibited the higher expression of Tim4 in PPs macrophages (Fig. S3A). Collectively, these data suggest that myeloid FoxO1 had an unexpected impact on Tim4^+^ intestinal macrophages in the gut.

We further assessed Tim4⁺ macrophages in the spleen and inguinal lymph nodes and observed increased proportions in FoxO1^M-KO^ septic mice, suggesting that FoxO1 deficiency induces systemic, rather than tissue-restricted, expansion of Tim4⁺ macrophages (Fig. 3G, H). Interestingly, the proportion of Tim4^+^ macrophages were also elevated in PPs of FoxO1^M-KO^ mice under non-septic conditions (Fig. S3B). Hence, our data demonstrate that myeloid FoxO1 deficiency systemically promotes the expansion of Tim4^+^ macrophages across both steady-state and septic conditions.

Previous studies have reported a reduction in CD8⁺ T cell percentages in both 24-hour CLP mice and septic patients 42-45. In addition to the increase in Tim4⁺ macrophages, FoxO1^M-KO^ septic mice showed elevated proportions of CD4⁺ and CD8⁺ T cells in PPs (Fig. S3C), with no significant changes in B cell populations (Fig. S3E). Myeloid FoxO1 deficiency also increased the proportion of activated CD8⁺ T cells (CD69⁺CD3⁺CD8⁺) in septic mice (Fig. S3C). Correlation analysis revealed a significant positive relationship between Tim4⁺ macrophages and CD8⁺ T cell frequency (Fig. S4). To further explore this interaction, we isolated Tim4⁺ peritoneal macrophages from FoxO1^FL/FL^ and FoxO1^M-KO^ septic mice and co-cultured them with CD8⁺ T cells activated with anti-CD3/CD28 microbeads. FoxO1 deletion in Tim4⁺ macrophages significantly enhanced CD8⁺ T cell proliferation (Fig. S3D), consistent with previous findings 46. Since CD8⁺ T cells mediate pathogen-specific immunity through targeted cytotoxic responses 47, these results suggest that myeloid FoxO1 deficiency promotes Tim4⁺ macrophage-driven enhancement of CD8⁺ T cell-mediated responses during sepsis. No differences were observed in lymphoid populations in PPs of non-septic FoxO1^FL/FL^ and FoxO1^M-KO^ mice (Fig. S3F, G).

### FoxO1 collaborates with Sin3a to inhibit the transcription of *Tim4* mRNA

**Figure 4:**
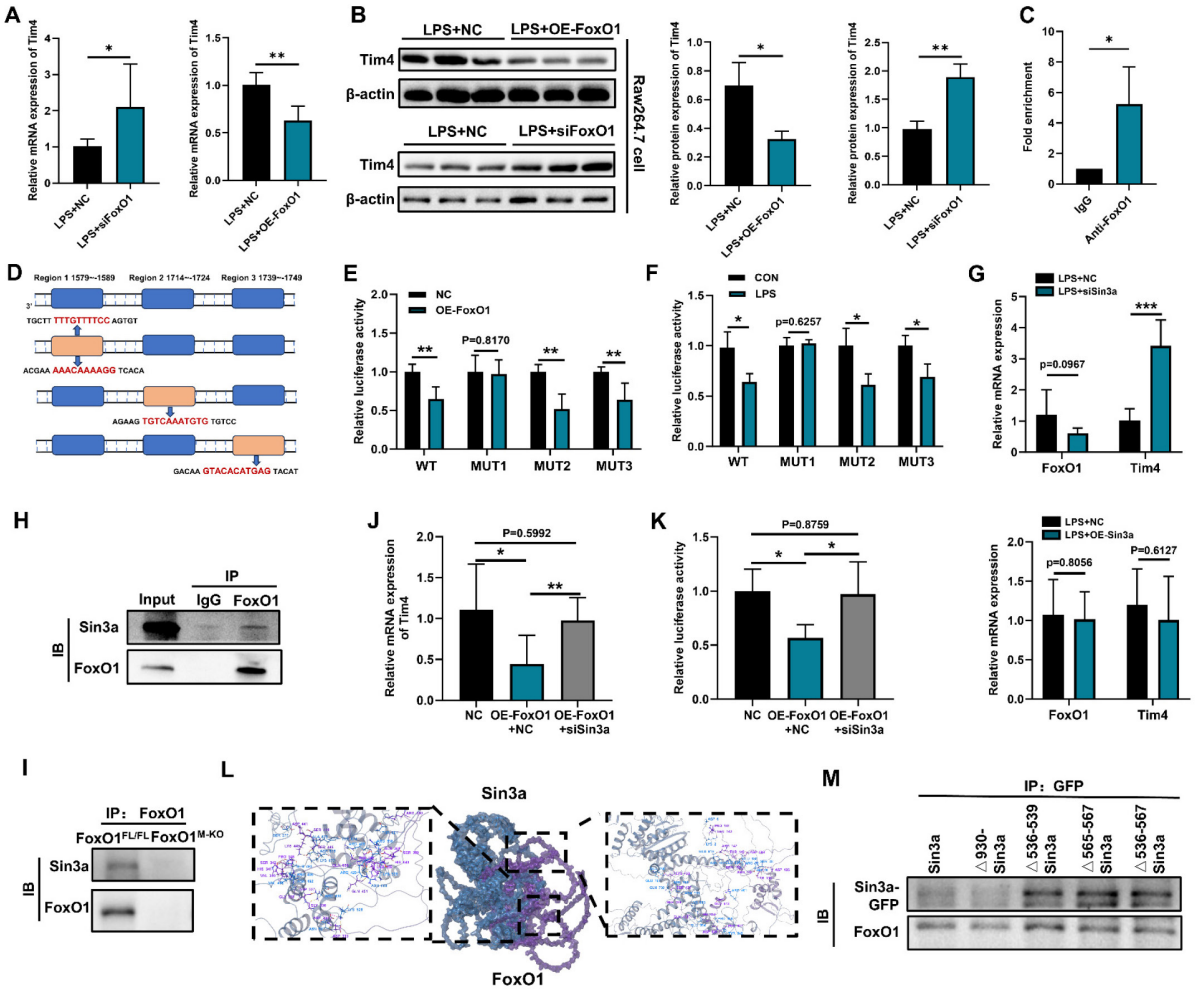
** FoxO1 collaborates with Sin3a to inhibit the transcription of *Tim4* mRNA. (A)** Relative mRNA expression of Tim4 in RAW264.7 cells with silencing FoxO1 (left) and overexpressing FoxO1 (right) under 48 h LPS treatment detected by qPCR (n=5). **(B)** Relative protein expression of Tim4 in RAW264.7 cells with silencing FoxO1 (up) and overexpressing FoxO1 (down) under 48 h LPS treatment detected by WB, the statistical results were showed as histograms (n=3). **(C)** Relative FoxO1 enrichment at the promoter of Tim4 was determined by ChIP-qPCR (n=3). **(D)** Predicted binding sites of FoxO1 on the Tim4 promoter. **(E)** Relative luciferase activity of Tim4 promoter WT and mutant site was detected after overexpressing FoxO1 48 h (n=5). **(F)** Relative luciferase activity of Tim4 promoter mutant site was detected under 24 h LPS treatment (n=5). **(G)** Relative mRNA expression of FoxO1 and Tim4 in RAW264.7 cells with silencing Sin3a (up) and overexpressing Sin3a (down) 48 h (n=5). **(H)** Co-immunoprecipitation (Co-IP) of Sin3a and FoxO1 in RAW264.7 cells under 24 h LPS treatment. **(I)** Co-IP of Sin3a and FoxO1 in Tim4^+^ primary macrophages from FoxO1^FL/FL^ and FoxO1^M-KO^ septic mice. **(J)** Relative mRNA expression of Tim4 in RAW264.7 cells with overexpressing FoxO1 and/or silencing Sin3a (n=5). **(K)** Relative luciferase activity of Tim4 promoter was detected overexpressing FoxO1 and/or silencing Sin3a (n=5). **(L)** The binding model of FoxO1 and Sin3a. **(M)** Co-IP of FoxO1 and Sin3a mutants in RAW264.7 cells under LPS treatment. Data represent the mean scores±SD. *P<0.05, **P<0.01, ***P<0.001.

Given the systemic expansion of Tim4⁺ macrophages observed with FoxO1 deficiency, we hypothesized that FoxO1 functions as a transcriptional regulator of Tim4. To explore this, FoxO1 small interfering RNA (siRNA) and overexpression plasmids were transfected into RAW264.7 cells treated with lipopolysaccharide (LPS) (Fig. S5A). Both in the presence and absence of LPS, FoxO1 knockdown increased Tim4 mRNA and protein expression (Fig. 4A, B, S5B), while FoxO1 overexpression reduced Tim4 expression (Fig. 4A, B, S5B). We then predicted potential FoxO1 binding motifs in the Tim4 promoter using the JASPAR database and designed specific primers for ChIP-qPCR analysis. FoxO1 was found to bind directly to the Tim4 promoter region spanning nucleotides 1559-1862, which contains three binding motifs (Fig. 4C, D). Luciferase reporter assays with site-specific mutations showed that FoxO1 significantly suppressed Tim4 promoter activity in the wild-type (WT) construct. Mutation of site 1 (MUT1) abolished this inhibitory effect, whereas mutations at sites 2 (MUT2) and 3 (MUT3) did not (Fig. 4E). Furthermore, LPS exposure inhibited Tim4 promoter activity in WT constructs, but this suppression was lost in MUT1-transfected cells, not in MUT2 or MUT3 (Fig. 4F). These findings indicated that FoxO1 directly binds to the Tim4 promoter at site 1579-1589, thereby repressing its transcription. This mechanism explains the increased Tim4 expression in macrophages following FoxO1 knockout.

Gene transcription is regulated through the coordinated activity of transcription factors and cofactors that assemble into transcriptional complexes 48. The transcriptional activity of FoxO1 is dynamically regulated by a variety of coregulators, including coactivators such as CBP/p300, corepressors like Sirt1, and post-translational modifying enzymes (AKT and JNK) 49, 50. Among these, SIN3 transcription regulator family member A (Sin3a) has been reported to interact with FoxO1 in primary hepatocytes to regulate glucokinase (Gck) transcription, contributing to hepatic glucose metabolism 51. However, Sin3a did not directly alter FoxO1 expression levels (Fig. 4G). Whether Sin3a is involved in regulating Tim4 transcription remained unclear. We found that silencing Sin3a significantly upregulated Tim4 mRNA expression, whereas overexpressing Sin3a had no effect (Fig. 4G). Transfection efficiency was confirmed (Fig. S5C, D). These findings suggest that Sin3a participates in Tim4 transcriptional regulation but is not its primary regulator. Co-immunoprecipitation assays confirmed that FoxO1 interacts with Sin3a in LPS-stimulated RAW264.7 cells (Fig. 4H). Besides, we isolated Tim4^+^ primary macrophages from FoxO1^FL/FL^ and FoxO1^M-KO^ septic mice, and found that the knockout of FoxO1 eliminated the detection of Sin3a in the immunoprecipitated (Fig. 4I). This suggests that Sin3a can interact with FoxO1 to regulate transcriptional activity in macrophages.

Overexpression of FoxO1 suppressed Tim4 mRNA expression, while Sin3a knockdown restored it (Fig. 4J). Moreover, Sin3a knockdown reversed FoxO1-induced inhibition of luciferase activity, indicating that Sin3a is a critical cofactor for FoxO1-mediated suppression of Tim4 transcription (Fig. 4K). Molecular docking analysis revealed strong binding stability between FoxO1 and Sin3a, with a PIPER pose energy of -2531.41 kcal/mol. Predicted interaction sites included two hydrogen bonds between FoxO1 (ARG153) and Sin3a (GLU930), and one hydrogen bond between FoxO1 (HIS640) and Sin3a (LYS536) (Fig. 4L). Sin3a has been reported to interact with multiple proteins, including histone deacetylase 1 (HDAC1) and AT-rich interaction domain 4B (ARID4B) 52, 53. To identify the FoxO1 binding region on Sin3a, we generated four plasmids expressing Sin3a mutants or truncated forms (Δ930-Sin3a, Δ536-539-Sin3a, Δ565-567-Sin3a, Δ536-567-Sin3a). Analysis showed that the binding sites were located within amino acids 536-567 of Sin3a (Fig. 4M). Collectively, these results demonstrate that the FoxO1-Sin3a complex is essential for inhibiting Tim4 transcription in macrophages during inflammation.

### FoxO1 aggravates Tim4^+^ macrophage glycolysis by MAP4K4 signaling

**Figure 5:**
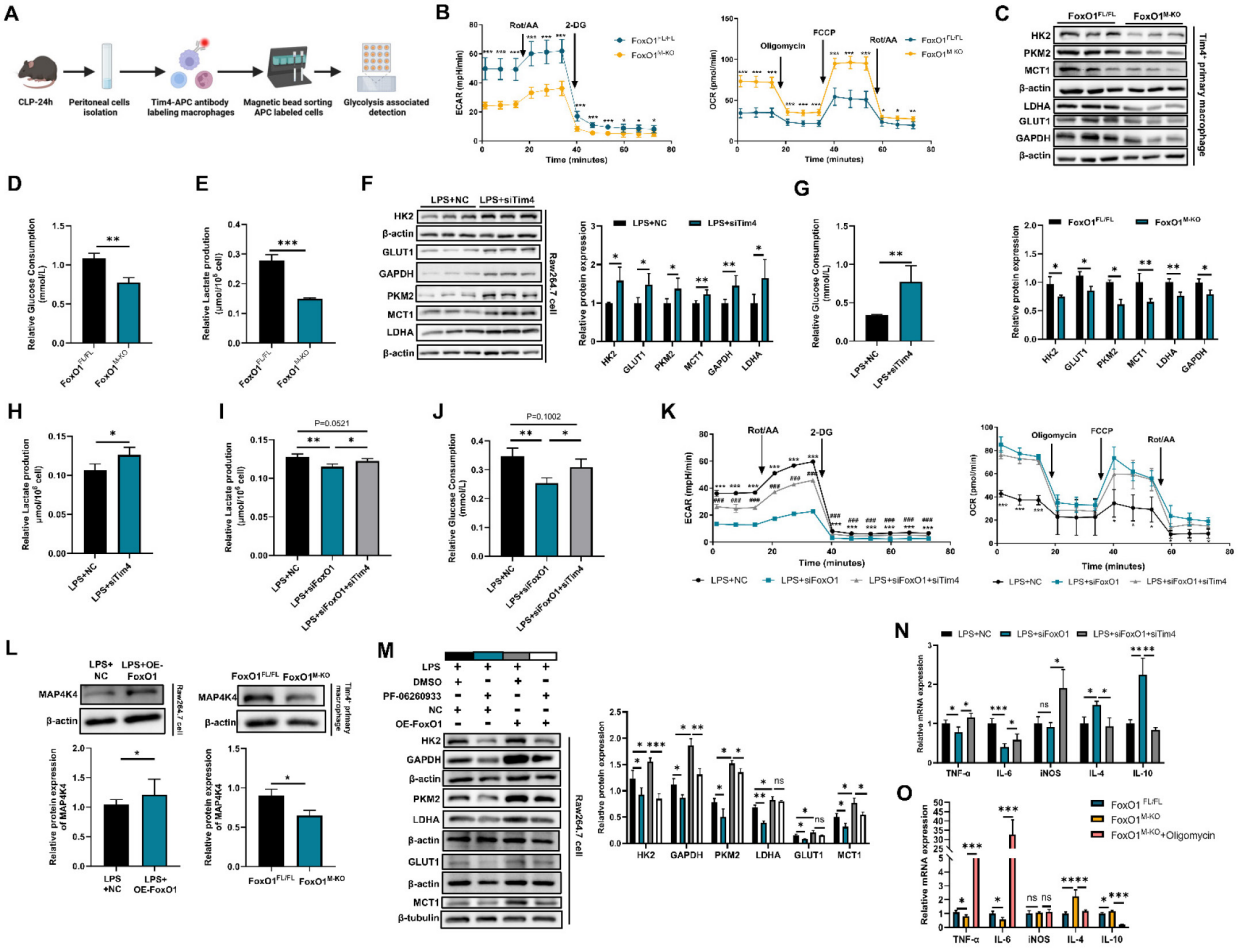
** FoxO1 aggravates Tim4^+^ macrophages glycolysis by MAP4K4 signaling. (A)** The procedure of isolating Tim4^+^ macrophages from CLP mice. **(B)** ECAR and OCR level of Tim4^+^ primary macrophages isolated from FoxO1^FL/FL^ and FoxO1^M-KO^ septic mice (n=6). **(C)** Relative protein expression of HK2, GLUT1, GAPDH, LDHA, PKM2 and MCT1 in Tim4^+^ primary macrophages isolated from FoxO1^FL/FL^ and FoxO1^M-KO^ septic mice, the statistical results were showed as histograms (n=3). **(D)** The consumption of glucose in Tim4^+^ primary macrophages isolated from FoxO1^FL/FL^ and FoxO1^M-KO^ septic mice (n=4). **(E)** The production of lactate in Tim4^+^ primary macrophages isolated from FoxO1^FL/FL^ and FoxO1^M-KO^ septic mice (n=4). **(F)** Relative protein expression of HK2, GLUT1, GAPDH, LDHA, PKM2 and MCT1 in RAW264.7 cells with silencing Tim4, the statistical results were showed as histograms (n=3). **(G)** The consumption of glucose in RAW264.7 cells with silencing Tim4 48 h (n=4). **(H)** The production of lactate in RAW264.7 cells with silencing Tim4 (n=4). **(I)** The consumption of glucose in RAW264.7 cells with silencing FoxO1 and/or silencing Tim4 (n=4). **(J)** The production of lactate in RAW264.7 cells with silencing FoxO1 and/or silencing Tim4 (n=4). **(K)** ECAR and OCR level of RAW264.7 cells with silencing FoxO1 and/or silencing Tim4 (n=6). **(L)** Relative expression of MAP4K4 in RAW264.7 cells with overexpressing FoxO1 (up) and Tim4^+^ primary macrophages isolated from FoxO1^FL/FL^ and FoxO1^M-KO^ septic mice (down) (n=3). **(M)** Relative protein expression of HK2, GLUT1, GAPDH, LDHA, PKM2 and MCT1 in RAW264.7 cells with DMSO/PF-06260933 (300 nM) and NC/OE-FoxO1 plasmid under LPS stimulation, the statistical results were showed as histograms (n=3). **(N)** Relative mRNA expression of TNF-α, IL-6, iNOS, IL-4 and IL-10 in RAW264.7 cells with silencing FoxO1 and/or silencing Tim4 (n=5). **(O)** Relative mRNA expression of TNF-α, IL-6, iNOS, IL-4 and IL-10 in Tim4^+^ primary macrophages isolated from FoxO1^FL/FL^ and FoxO1^M-KO^ septic mice and/or Oligomycin treatment (n=4). Data represent the mean scores±SD. *P<0.05, **P<0.01, ***P<0.001.

To investigate the impact of increased Tim4⁺ intestinal macrophages in FoxO1^M-KO^ septic mice, we analyzed transcriptomic data from PPs macrophages. Bonnardel et al. identified only 55 differentially expressed genes (DEGs) between Tim4⁺ and Tim4⁻ macrophages in PPs 54. GO analysis indicated that “regulation of glucose metabolic process” was a key biological pathway enriched in Tim4⁺ macrophages (Fig. S6A). Since glycolysis is closely linked to FoxO1 activity 51, 55, we examined whether FoxO1 influences glycolytic metabolism in these cells. Silencing FoxO1 in RAW264.7 cells reduced the expression of glycolytic enzymes, including hexokinase 2 (HK2), M2-type pyruvate kinase (PKM2), lactate dehydrogenase A (LDHA), and glyceraldehyde-3-phosphate dehydrogenase (GAPDH), as well as transporters such as glucose transporter type 1 (GLUT1) and monocarboxylate transporter 1 (MCT1) under LPS stimulation. Conversely, FoxO1 overexpression enhanced their expression (Fig. S6B, C). To further confirm these findings, we enriched Tim4⁺ macrophages from peritoneal cells using magnetic bead sorting, the sorting efficiency has been verified (Fig. 5A, Fig. S6D). We assessed their metabolic state by measuring the extracellular acidification rate (ECAR) and oxygen consumption rate (OCR). FoxO1 knockout significantly reduced the glycolytic capacity (ECAR) of Tim4⁺ macrophages, while increasing their oxygen consumption (Fig. 5B). Protein levels of glycolysis-related enzymes were also reduced in Tim4⁺ macrophages derived from FoxO1^M-KO^ septic mice (Fig. 5C, Fig. S6E). Moreover, glucose consumption and lactate production were significantly lower in FoxO1^M-KO^ mice, indicating attenuated glycolytic activity (Fig. 5D, E).

Knocking down Tim4 increased the expression of glycolysis-associated proteins, as well as glucose consumption and lactate production in RAW264.7 cells under LPS stimulation, indicating that Tim4 contributes to maintaining macrophage glycolytic activity (Fig. 5F-H). To further investigate this, we silenced Tim4 in FoxO1 knockdown macrophages and observed enhanced glucose uptake and lactate production under LPS treatment, along with increased ECAR levels (Fig. 5I-K). Notably, Tim4 silencing suppressed OCR levels (Fig. 5K). These findings suggest that Tim4 is critical for FoxO1-mediated regulation of macrophage glycolysis. GO analysis identified mitogen-activated protein kinase kinase kinase kinase 4 (MAP4K4) as a key regulator of glucose metabolism (Fig. S6A). MAP4K4 has been reported to enhance glycolysis in human hepatocytes through the JNK signaling pathway 56.

Our RNA sequencing data showed a significant positive correlation between FoxO1 and MAP4K4 expression in PPs macrophages from CLP mice (Fig. S6F). Moreover, MAP4K4 protein expression was reduced in Tim4⁺ macrophages from FoxO1^M-KO^ septic mice, whereas FoxO1 overexpression increased MAP4K4 levels in RAW264.7 cells under LPS stimulation (Fig. 5L). To further investigate the potential role of MAP4K4 in FoxO1-mediated macrophage glycolysis, we employed its selective small-molecule inhibitor, PF-06260933 57, 58. Under LPS stimulation, PF-06260933 significantly inhibited the expression of key glycolysis-related enzymes in macrophages. FoxO1 overexpression markedly enhanced the expression of these glycolytic enzymes. Notably, PF-06260933 treatment effectively reversed the FoxO1-driven upregulation and partially suppressed glycolytic activity (Fig. 5M). Together, these findings indicate that FoxO1 regulates glycolysis in Tim4⁺ macrophages through MAP4K4 signaling.

Macrophages undergo metabolic reprograming to adjust their energy utilization and respond to inflammatory stimuli 59. Following the increased glycolytic level of RAW264.7 cells, silencing Tim4 increased the expression of pro-inflammatory genes (TNF-α, IL-6, iNOS), and decreased anti-inflammatory genes (IL-4, IL-10) when FoxO1 was knocked down under LPS stimulation (Fig. 5N).

We also used oligomycin, a glycolysis activator, to further enhance glycolytic flux 60. Compared with Tim4^+^ macrophages in FoxO1^FL/FL^ septic mice, the pro-inflammatory genes (TNF-α, IL-6) were down-regulated and the anti-inflammatory genes (IL-4, IL-10) were up-regulated in FoxO1^M-KO^ mice, and oligomycin reversed these effects (Fig. 5O). Overall, these results demonstrate that FoxO1 enhances glycolysis in Tim4⁺ macrophages through MAP4K4 signaling, thereby driving pro-inflammatory responses in sepsis.

### Macrophage FoxO1 is a therapeutic target for septic intestinal injury

**Figure 6:**
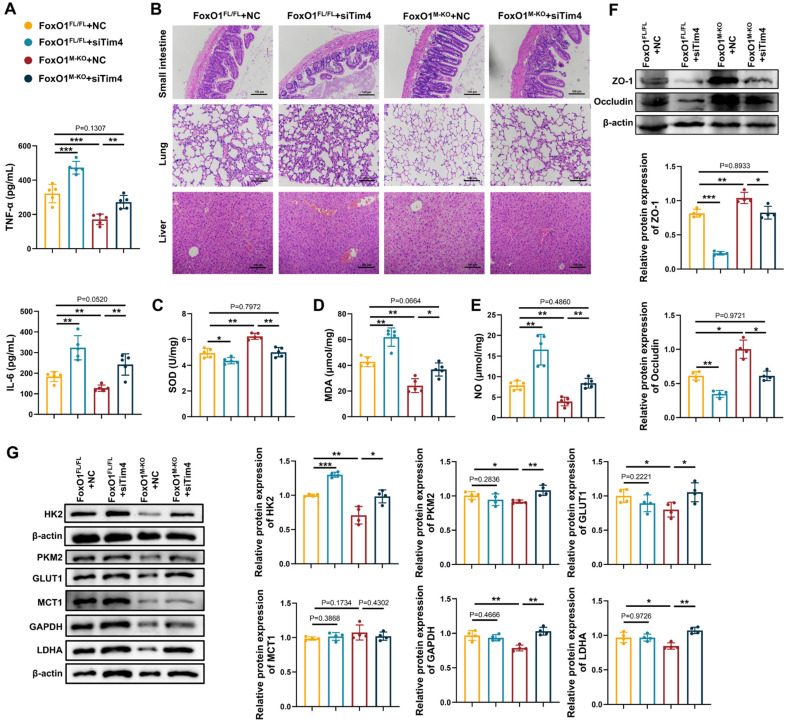
** Macrophage FoxO1 is a therapeutic target for septic intestinal injury. (A)** The level of TNF-α and IL-6 in serum from FoxO1^FL/FL^ and FoxO1^M-KO^ CLP mice with adoptively transferring knock-down Tim4 macrophages determined by ELISA (n=5). **(B)** Small intestine, lung and liver histopathological evaluation by HE staining. Representative images of these tissues from FoxO1^FL/FL^ and FoxO1^M-KO^ CLP mice with adoptively transferring knock-down Tim4 macrophages (n=5). **(C-E)** The enzyme activity of SOD, the level of MDA and NO in small intestine tissues from FoxO1^FL/FL^ and FoxO1^M-KO^ CLP mice with adoptively transferring knock-down Tim4 macrophages (n=5). **(F)** Relative protein expression of ZO-1 and Occludin in small intestine tissues from FoxO1^FL/FL^ and FoxO1^M-KO^ CLP mice with adoptively transferring knock-down Tim4 macrophages, the statistical results were showed as histograms (n=4). **(G)** Relative protein expression of HK2, GLUT1, GAPDH, LDHA, PKM2 and MCT1 in PPs from FoxO1^FL/FL^ and FoxO1^M-KO^ CLP mice with adoptively transferring knock-down Tim4 macrophages (n=4). Data represent the mean scores±SD. *P<0.05, **P<0.01, ***P<0.001.

To further assess the role of Tim4⁺ macrophages in sepsis, we investigated whether adoptive transfer of Tim4-knockdown macrophages would affect disease progression in FoxO1^M-KO^ septic mice. Since Tim4 is not universally expressed in bone marrow derived cells 61-63, we transfected Tim4-siRNA into peritoneal macrophages for adoptive transfer into FoxO1^M-KO^ septic mice, with knockout efficiency confirmed (Fig. S7). Adoptive transfer of Tim4-knockdown macrophages increased serum TNF-α and IL-6 levels in both FoxO1^FL/FL^ and FoxO1^M-KO^ septic mice (Fig. 6A). Tissue damage, including injury to the small intestine, lung, and liver, was exacerbated in both groups following the injection of Tim4-knockdown macrophages (Fig. 6B). Evaluation of tight junction proteins, along with altered SOD, MDA, and NO levels in the small intestine, revealed that Tim4-knockdown macrophages induced significant intestinal barrier damage and oxidative stress (Fig. 6C-F). The protective effects observed in FoxO1^M-KO^ mice were completely reversed by the adoptive transfer of Tim4-knockdown macrophages in septic mice. In addition, glycolytic proteins expression in PPs was elevated following adoptive transfer, indicating that enhanced glycolysis was associated with heightened inflammation (Fig. 6G). These findings demonstrate that the protective effects of myeloid FoxO1 deficiency is dependent on the presence of Tim4⁺ macrophages, identifying macrophage FoxO1 as a therapeutic target for septic intestinal injury.

### HSBD ameliorates septic intestinal damage inhibiting macrophage FoxO1

**Figure 7:**
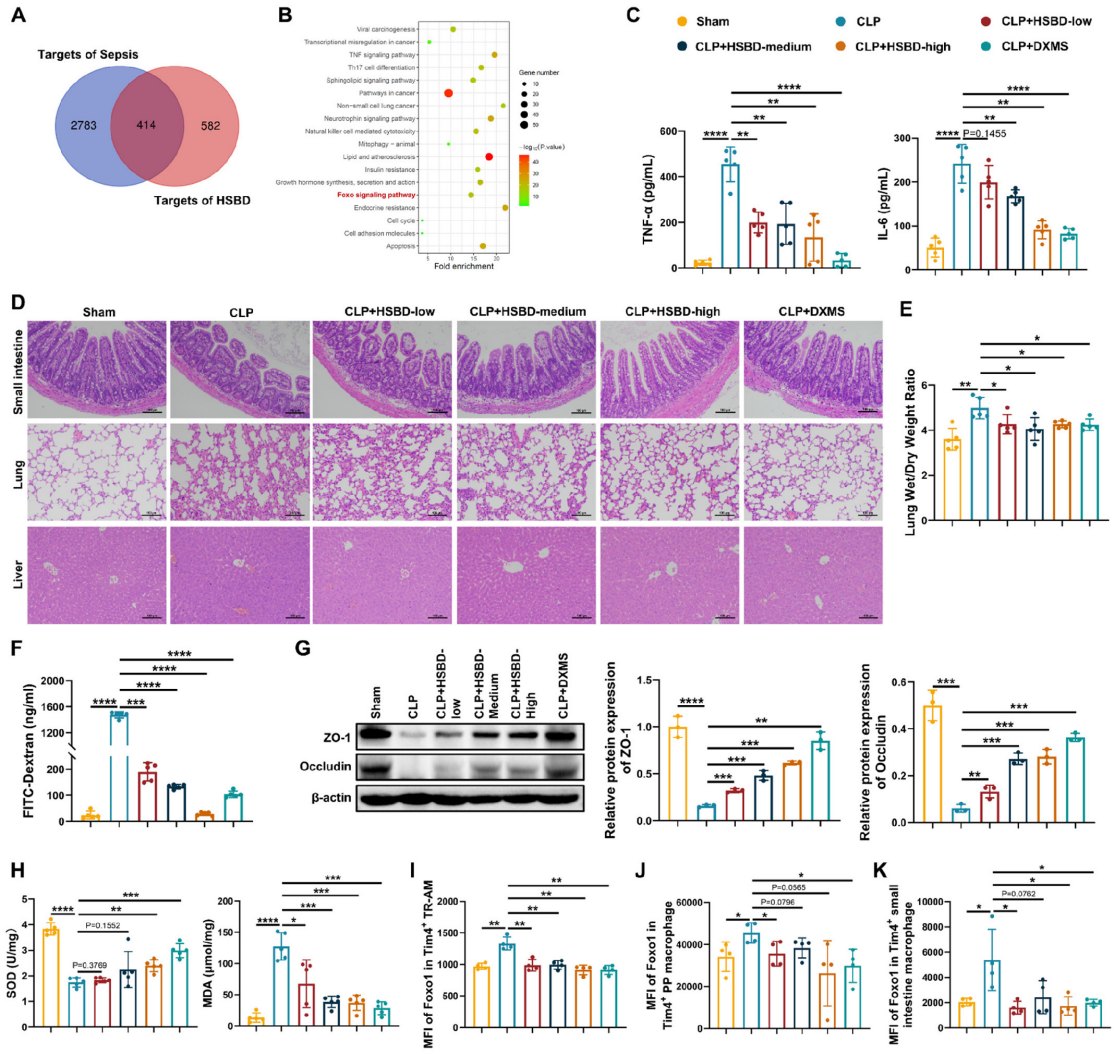
** HSBD ameliorates septic intestinal damage inhibiting macrophage FoxO1. (A)** Venn diagram of targets for sepsis and traditional Chinese medicine formula HSBD. **(B)** KEGG analysis according to top 100 predicted targets for HSBD in sepsis. **(C)** The level of TNF-α and IL-6 in serum from sham, CLP, CLP+HSBD (low: 0.7 g/kg; medium: 1.4 g/kg; high: 2.8 g/kg), CLP+DXMS groups (n=5). **(D)** Small intestine, lung and liver histopathological evaluation by HE staining. Representative images of these tissues from sham, CLP, CLP+HSBD (low, medium, high), CLP+DXMS groups (n=5). **(E)** The ratio of lung wet and dry weight in sham, CLP, CLP+HSBD (low, medium, high), CLP+DXMS mice (n=5). **(F)** The fluorescence of FITC-Dextran in serum from sham, CLP, CLP+HSBD (low, medium, high), CLP+DXMS mice (n=5). **(G)** Relative protein expression of ZO-1 and Occludin in small intestine tissues from sham, CLP, CLP+HSBD (low, medium, high), CLP+DXMS mice, the statistical results were showed as histograms (n=3). **(H)** The enzyme activity of SOD, the level of MDA in small intestine tissues from sham, CLP, CLP+HSBD (low, medium, high), CLP+DXMS mice (n=5). **(I-K)** MFI of FoxO1 in Tim4^+^ tissue resident alveolar macrophages, Tim4^+^ PP macrophages, Tim4^+^ small intestinal macrophages from sham, CLP, CLP+HSBD (low, medium, high), CLP+DXMS mice (n=4). Data represent the mean scores±SD. *P<0.05, **P<0.01, ***P<0.001, ****P<0.0001.

A recent study reported that the Huashi Baidu formula (HSBD), a traditional Chinese medicine formula developed by the Chinese Academy of Traditional Chinese Medicine, alleviated symptoms such as fever, cough, and fatigue in patients with COVID-19 27. HSBD was effective in treating sepsis-induced acute lung injury through suppressing cytokine storms and mitigating endothelial injury, suggesting its therapeutic potential for septic intestinal injury 27, 64. Therefore, we first performed network pharmacology analysis to evaluate the effect of HSBD on FoxO1 in sepsis (Fig. S8A). HSBD formula consists of 14 traditional Chinese medicine, such as Cangzhu, Fuling, Shigao, Huangqi, Banxia 65. Xu et al. further identified 60 prototype compounds in HSBD 66. A total of 996 targets for these compounds were identified from SwissTargetPrediction, and 3,197 sepsis-related targets were retrieved from the GeneCards database. The intersection comprised 414 potential targets for HSBD in sepsis (Fig. 7A). A protein-protein interaction (PPI) network was constructed using the STRING database with a medium confidence threshold (>0.4). The network was visualized in Cytoscape, and the top 100 targets, based on degree score, were selected for KEGG pathway analysis (Fig. S8B). FoxO signaling was among the enriched pathways, highlighting its potential involvement in HSBD mechanisms (Fig. 7B).

To evaluate the therapeutic effects of HSBD on macrophage FoxO1 and septic intestinal injury, we administered different doses of HSBD by gavage to CLP mice, with dexamethasone (DXMS) serving as a positive control. Both HSBD and DXMS significantly reduced TNF-α and IL-6 levels in peripheral blood compared with the CLP group (Fig. 7C). HE staining of the small intestine, lung, and liver showed that HSBD alleviated tissue damage in a dose-dependent manner (Fig. 7D). The lung wet/dry weight ratio was reduced in both CLP+HSBD and CLP+DXMS groups, indicating decreased pulmonary edema (Fig. 7E). To further assess the effects of HSBD on septic intestinal injury, intestinal barrier permeability and oxidative stress were evaluated. HSBD and DXMS significantly reduced FITC-Dextran leakage into peripheral blood and increased the expression of tight junction proteins in the small intestine (Fig. 7F, G). Both treatments also elevated SOD levels and decreased MDA levels in the small intestine of CLP mice (Fig. 7H). These findings suggest that HSBD exerts potent therapeutic effects in sepsis, particularly by reducing intestinal injury. We then used flow cytometry to measure FoxO1 expression in Tim4⁺ macrophages from CLP mice treated with HSBD or DXMS. Macrophages isolated from bronchoalveolar lavage fluid (BALF), intestinal lamina propria, and PPs were analyzed. Macrophages in BALF were categorized into tissue-resident alveolar macrophages (TR-AMs), monocyte-derived alveolar macrophages (Mo-AMs), or interstitial macrophages (IMs) 67. TR-AMs are often reduced during acute inflammation, and their efferocytotic activity is associated with anti-inflammatory and pro-repair phenotypes 68, 69. We focused on Tim4⁺ cells in TR-AMs, with gating strategies shown in Fig. S9. Both HSBD and DXMS downregulated FoxO1 expression in Tim4⁺ TR-AMs, Tim4⁺ PP macrophages, and Tim4⁺ intestinal lamina propria macrophages (Fig. 7I-K). These findings indicate that HSBD mitigates septic intestinal injury by inhibiting macrophage FoxO1.

## Discussion

Our study demonstrated that myeloid-specific FoxO1 knockout alleviates intestinal injury in sepsis and that FoxO1, as a key transcription factor, regulates Tim4⁺ macrophages during septic intestinal injury. FoxO1 suppressed Tim4 transcription in macrophages and enhanced glycolysis in Tim4⁺ macrophages in septic mice. This dual role of FoxO1 not only explains the observed changes in cell populations but also clarifies the anti-inflammatory effects of Tim4⁺ macrophages in FoxO1^M-KO^ septic mice. These results highlight FoxO1 as a critical regulator of innate immune responses during sepsis. Furthermore, we found that HSBD effectively reduced FoxO1 expression in Tim4⁺ macrophages, thereby alleviating septic intestinal injury. This suggests that HSBD may serve as a promising therapeutic strategy targeting FoxO1 in sepsis-related conditions. Collectively, our results establish macrophage FoxO1 as a novel therapeutic target for septic intestinal injury and provide mechanistic evidence supporting the clinical potential of HSBD in treating sepsis-induced inflammation.

FoxO1 plays a crucial role in metabolic regulation across multiple cell types. For example, FoxO1 knockdown restores glycolytic capacity and promotes DNA repair in endothelial cells 70. However, FoxO1 overexpression suppresses endothelial metabolic activity and limits cell proliferation 71. The deficiency of FoxO1 in astrocytes disrupts glucose regulation, contributing to diet-induced obesity 72. Thus, FoxO1 participated in diverse biological processes by modulating cell metabolism. Otherwise, glycolysis is dramatically associated with the immune functions of macrophages, including the production of pro-inflammatory cytokines, chemotaxis and polarization of macrophages 73, 74. Calciumloside E inhibited glycolysis-mediated M1 macrophage polarization to treat atherosclerosis 75. Zhong et al. found 2-DG (the inhibitor of glycolysis) diminished NLRP3 inflammasome activation of macrophages in acute lung injury 76. Our study characterized FoxO1 as a major regulator of metabolic reprogramming in Tim4^+^ macrophages, which further regulating macrophage activation in septic intestinal injury.

Tim4^+^ macrophages are originated from both embryonic precursors and adult bone marrow derived monocytes. Tim4 is a scavenger receptor in the macrophages for phagocytosis of apoptotic cells, and some studies have explained other vital functions of Tim4^+^ macrophages in the gut. For example, Tim4^+^CD4^+^ macrophages in the colon of colitis mice showed immunosuppressive capacity to inhibit T cells proliferation and induce T cells apoptosis 77. Diet-induced obesity decreased the quantities of Tim4^+^CD4^+^ macrophages in ileum and colon, along with the alternation of intracellular cytokines IL-10 and TNF 78. Tim4^+^ macrophages substantially modulate inflammation in the gut, and this regulatory capacity is largely governed by their metabolic diversity. Tim4^+^ colonic macrophages showed enhanced fatty acid uptake alongside reduced fatty acid synthesis after antibiotic exposure 79. Heieis et al. reported that that Tim4⁺ macrophages in the intestinal lamina propria express metabolic proteins that differ markedly from those in Tim4⁻ macrophages, reflecting metabolic heterogeneity within the Tim4⁺ macrophage population in the intestine 80. However, the metabolic determinants of Tim4⁺ macrophage function have remained unclear. We found the decreased glycolytic ability of Tim4^+^ intestinal macrophages were markedly correlated with the down-regulated expression of pro-inflammatory factors and remission of intestinal injury. Tim4 have been verified as a switch to induce M2 macrophages polarization in obesity mice 81. Furthermore, some studies have showed that deficiency of FoxO1 resulted in an alternatively activated M2 phenotype of macrophages 55. We also observed a reduction of M2 macrophages in PPs of CLP mice based on immune infiltration analysis. Together, these findings suggest that Tim4⁺ macrophages exert anti-inflammatory effects resembling the M2 phenotype, mediated in part through FoxO1-dependent glycolysis during sepsis. The inflammatory role of Tim4⁺ macrophages was further confirmed by the exacerbation of sepsis in CLP mice following adoptive transfer of Tim4-knockdown macrophages. Additionally, reversal of FoxO1 knockdown effects on glycolysis in RAW264.7 cells using Tim4-siRNA induced modest but significant changes, suggesting that while Tim4 contributes to FoxO1-mediated regulation of macrophage glycolysis, it is not the sole mediator.

We found that FoxO1 interacted with Sin3a to negatively regulate Tim4 transcription. This finding is part of a broader regulatory context, as FoxO1's transcriptional activity is known to be modulated by multiple factors. For example, it has been reported that FoxO1 can cooperatively regulate downstream gene promoters with the transcription factor CEBPB. We also demonstrated that FoxO1 directly bind to CEBPB in macrophages following LPS stimulation (Fig. S5E), revealing the complex molecular regulatory network in which FoxO1 acts as a pivotal transcription factor in sepsis. Furthermore, FoxO1's transcriptional regulatory functions have been widely documented in processes such as cell cycle arrest, apoptosis, DNA repair, and cell chemotaxis. For instance, FoxO1 inhibited the increase of nuclear factor-kappaB (NF-κB) activity during endoplasmic reticulum stress, leading to an exacerbation of macrophage apoptotic response 82. FoxO1 activity can be regulated through its mRNA and protein modifications, making it a therapeutic target for conditions like cancer, wound healing, and diabetes. Several small molecule compounds and drugs have been developed to selectively inhibit FoxO1. For example, AS1842856 blocked FoxO1 activity, lowering plasma glucose levels 83. Puerarin could antagonize FoxO1 activation to suppress macrophage polarization towards M1 phenotype, thereby ameliorating nephritis 84. Our study revealed that HSBD, a traditional Chinese medicine compound approved by the NMPA in 2021, targets FoxO1 in Tim4⁺ macrophages, reducing septic organ damage, especially in the intestine. HSBD consists of 14 Chinese herbs and has multiple potential targets in various diseases 85. Although FoxO1 is not the sole molecular target of HSBD in sepsis, the marked inhibitory effect of HSBD on macrophage FoxO1 in septic mice suggests a promising therapeutic relationship that warrants further mechanistic investigation. Further elucidation of this relationship may not only clarify the molecular basis of HSBD's efficacy but also facilitate its clinical application in sepsis management.

In summary, our study elucidates the immunoregulatory role of FoxO1 in septic intestinal injury, highlighting the contribution of Tim4⁺ macrophages to the inflammatory process. The regulatory effect of FoxO1 on Tim4 transcription underscores its importance in modulating macrophage function within the inflammatory microenvironment. Our findings suggest that targeting FoxO1 in macrophages may represent an effective therapeutic strategy for sepsis-associated intestinal injury.

## Supplementary Material

Supplementary figures and table.

## Abbreviations

FoxO1: Forkhead Box O1
PPs: Peyer's patches
HSBD: Huashi Baidu formula
ICU: Intensive care unit
mTORC: mechanistic target of rapamycin complex
TLR4: Toll-like receptor 4
SPF: Specific pathogen-free
Lyz2: Lysozyme 2
CLP: Cecal ligation and puncture
NMPA: National Medical Products Administration
DXMS: Dexamethasone
LPS: Lipopolysaccharide
ACK: Ammonium-Chloride-Potassium
PBS: Phosphate buffered saline
NC: Negative control
TNF-α: Tumor necrosis factor-α
SOD: Superoxide dismutase
MDA: Malondialdehyde
NO: Nitric oxide
WT: Wild-type
ChIP: Chromatin immunoprecipitation
OCR: Oxygen consumption rate
ECAR: Extracellular acidification rate
ELISA: Enzyme-linked immunosorbent assay
DEG: Differentially expressed gene
HE: Hematoxylin/eosin
PFA: Paraformaldehyde
MFI: Mean fluorescence intensity
IL-6: Interleukin-6
GO: Gene Ontology
Tim4: T cell immunoglobulin and mucin domain containing 4
siRNA: small interfering RNA
Sin3a: SIN3 transcription regulator family member A
Gck: Glucokinase
HDAC1: Histone deacetylase 1
ARID4B: AT-rich interaction domain 4B
HK2: Hexokinase 2
PKM2: M2-type pyruvate kinase
LDHA: Lactate dehydrogenase A
GAPDH: Glyceraldehyde-3-phosphate dehydrogenase
GLUT1: Glucose transporter type 1
MCT1: Monocarboxylate transporter 1
MAP4K4: Mitogen-activated protein kinase kinase kinase kinase 4
PPI: Protein-protein interaction
BALF: Bronchoalveolar lavage fluid
NF-κB: Nuclear factor-kappaB
