# Supplementary material for: Targeting Myeloid FoxO1 Ameliorates Sepsis-induced Intestinal Injury by Modulating Tim4+ Macrophage Glycolysis
Source: Int J Biol Sci. 2026 Jan 1;22(1):239–57. doi: 10.7150/ijbs.119052 (PMC12681812; doi:10.7150/ijbs.119052)
Supplement: Supplementary file 1 — Supplementary figures and table. [file ijbsv22p0239s1.pdf]

Supplementary material 1

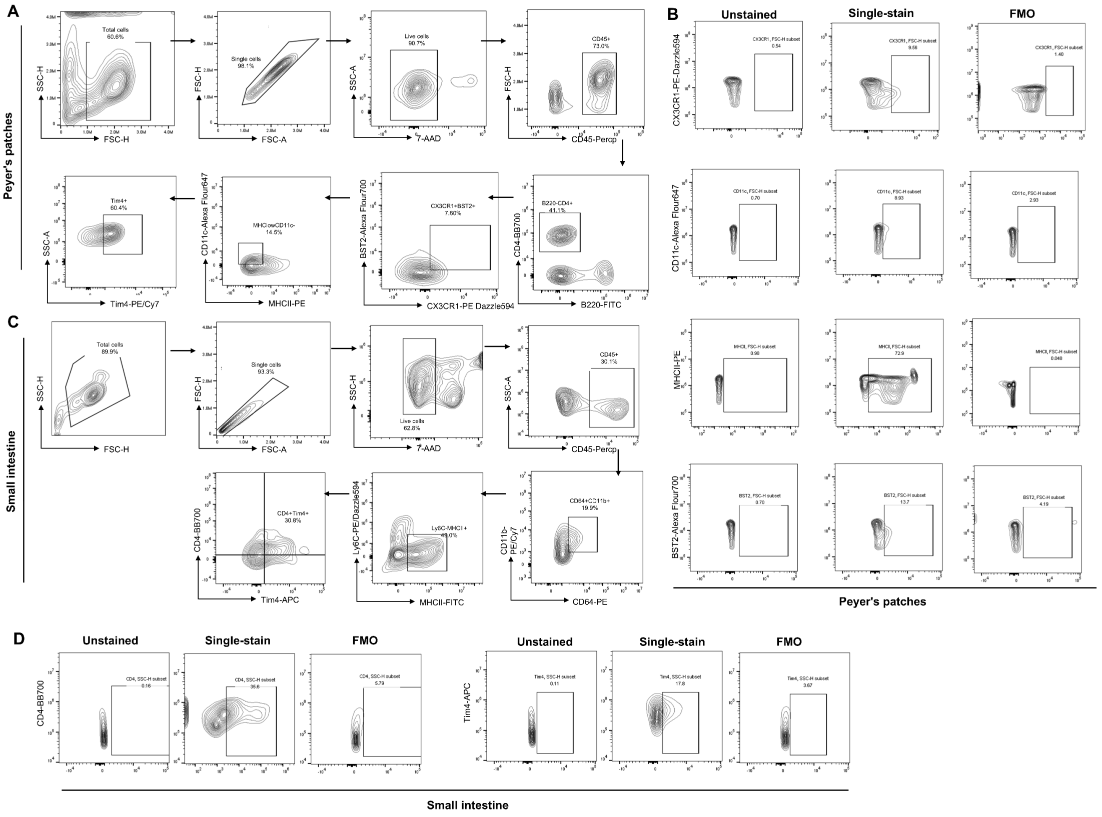

Fig. S1 (A, C) The gating strategy of Tim4^{+} macrophages in PPs and small intestine. (B) The unstained, single-stain and FMO tube in Tim4^{+} macrophages from PPs. (D) The unstained, single-stain and FMO tube in Tim4^{+} macrophages from small intestine.

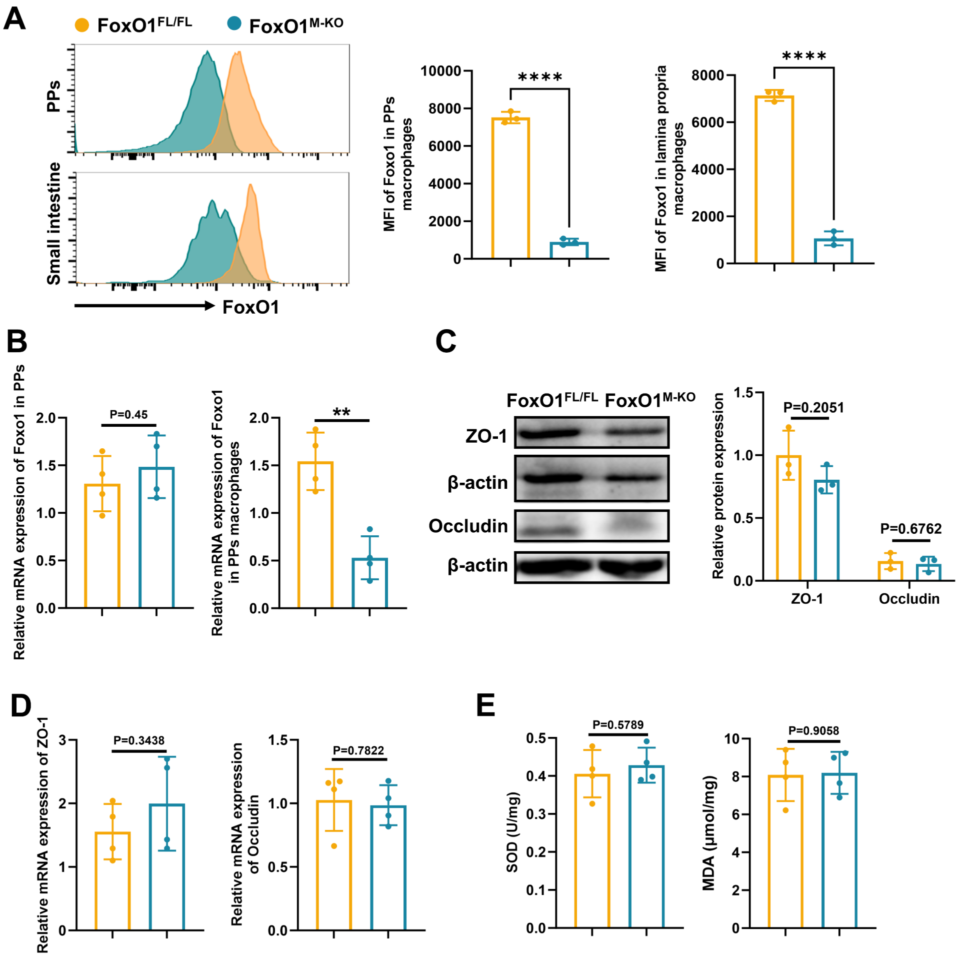

Fig. S2 (A) MFI of FoxO1 in PPs and lamina propria macrophages in FoxO1$^{FL/FL}$ and FoxO1^{M-KO} mice. (B) Relative mRNA expression of FoxO1 in PPs (left) and PPs macrophages (right). (C) Relative mRNA expression of ZO-1 and occludin in small intestine from FoxO1$^{FL/FL}$ and FoxO1^{M-KO} mice. (D) Relative protein expression of ZO-1 and occludin in small intestine from FoxO1$^{FL/FL}$ and FoxO1^{M-KO} mice. (E) The level of SOD and MDA in small intestine from FoxO1$^{FL/FL}$ and FoxO1^{M-KO} mice. Data represent the mean scores±SD. **P<0.01.

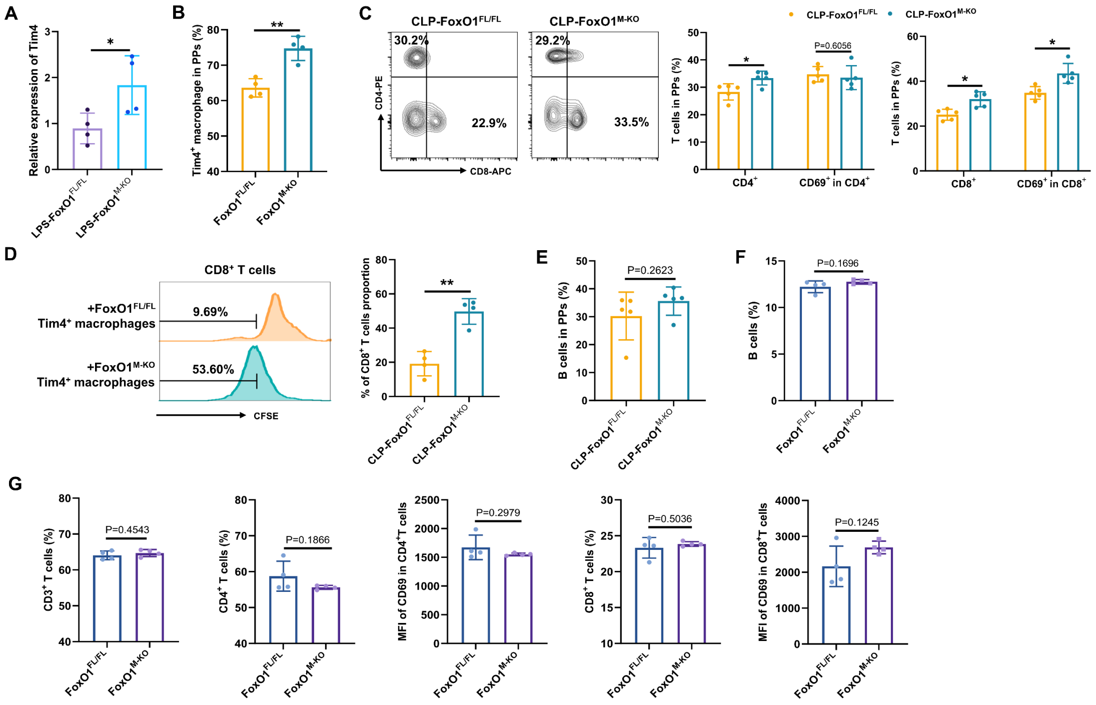

Fig. S3 (A) The mRNA expression of Tim4 in PPs macrophages from LPS-induced FoxO1$^{FL/FL}$ and FoxO1^{M-KO} mice. (B) The frequency of Tim4^{+} macrophages in PPs from FoxO1$^{FL/FL}$ and FoxO1^{M-KO} mice. (C) The proportion of CD4^{+} and CD8^{+} T cells in PPs from FoxO1$^{FL/FL}$ and FoxO1^{M-KO} septic mice were determined by flow cytometry. (D) Tim4^{+} peritoneal macrophages from FoxO1$^{FL/FL}$ or FoxO1^{M-KO} septic mice were co-cultured with splenic CD8^{+} T cells. CD8^{+} T cell proliferation was assessed by CFSE staining. (E)The proportion of B cells in PPs from FoxO1$^{FL/FL}$ and FoxO1^{M-KO} septic mice. (F) The proportion of CD4^{+} and CD8^{+} T cells in PPs from FoxO1$^{FL/FL}$ and FoxO1^{M-KO} normal mice were determined by flow cytometry. (G) The proportion of B cells in PPs from FoxO1$^{FL/FL}$ and FoxO1^{M-KO} normal mice were determined by flow cytometry. Data represent the mean scores±SD. *P<0.05, **P<0.01.

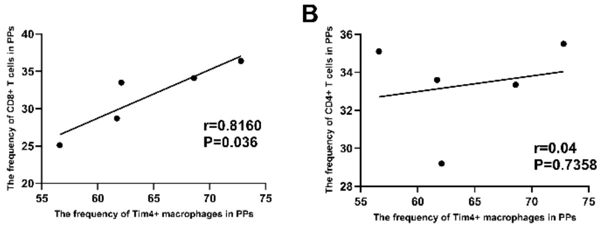

Fig. S4 The correlation of Tim4^{+} macrophages and CD4^{+}, CD8^{+} T cells in PPs from FoxO1$^{M\text{-}KO}$ septic mice.

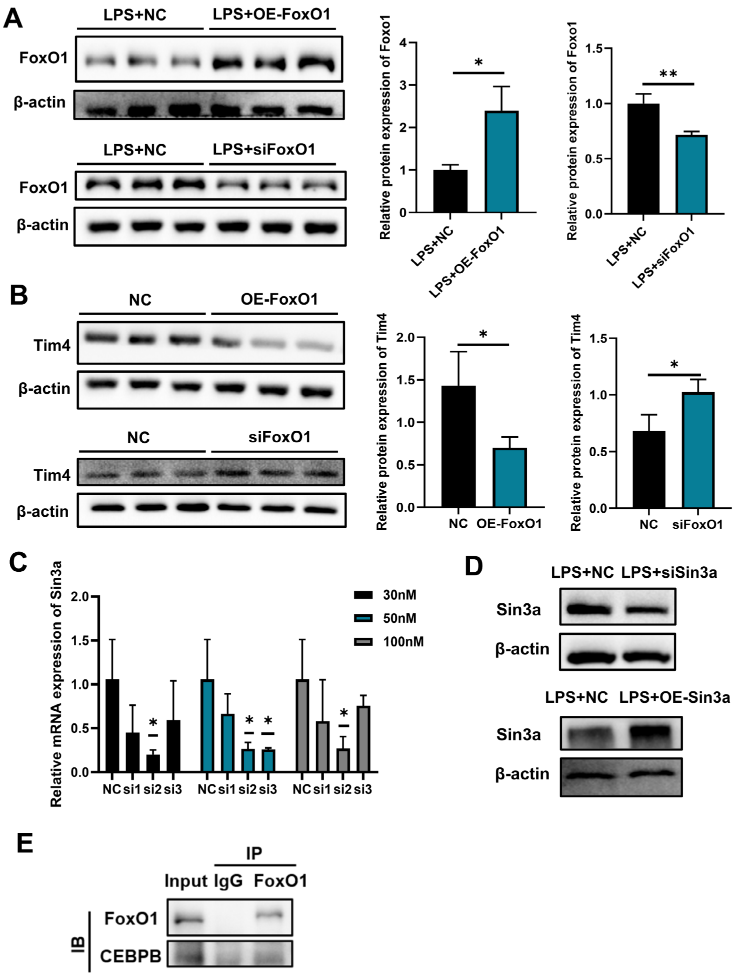

Fig. S5 (A) Relative protein expression of FoxO1 in RAW264.7 cells with overexpressing FoxO1 or silencing FoxO1 under LPS treatment. (B) Relative protein expression of Tim4 in RAW264.7 cells with overexpressing FoxO1 or silencing FoxO1 without LPS treatment. (C) Relative mRNA

expression of Sin3a in RAW264.7 cells with silencing Sin3a. (D) Relative protein expression of Sin3a in RAW264.7 cells with silencing and overexpressing Sin3a. (E) Co-IP of CEBPB and FoxO1 in RAW264.7 cells under 24 h LPS treatment. Data represent the mean scores±SD. *P<0.05, **P<0.01.

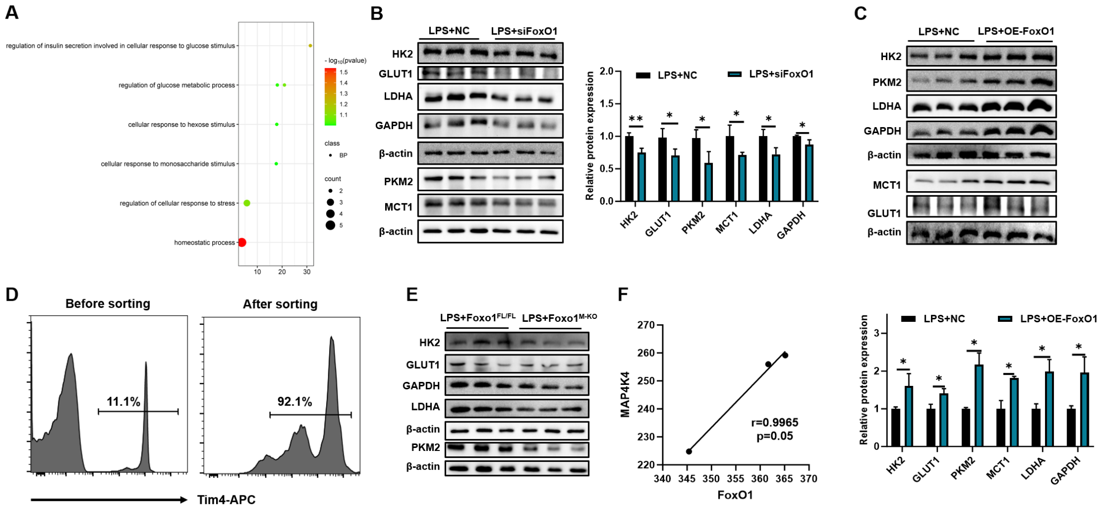

Fig. S6 (A) GO analysis of DEGs between Tim4+ and Tim4- macrophages in PPs (Data from GSE65514). (B, C) Relative protein expression of HK2, GLUT1, GAPDH, LDHA, PKM2 and MCT1 in RAW264.7 cells with overexpressing FoxO1 under LPS treatment. (D) The sorting efficiency of Tim4+ cells detected by flow cytometry. (E) Relative protein expression of HK2, GLUT1, GAPDH, LDHA, PKM2 in Tim4+ primary macrophages isolated from LPS-induced FoxO1$^{FL/FL}$ and FoxO1$^{M\text{-}KO}$ mice. (F) The correlation of FoxO1 and MAP4K4 in the RNA sequencing data based on PPs macrophages from CLP mice. Data represent the mean scores±SD. *P<0.05.

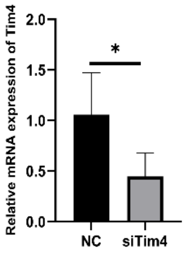

Fig. S7 Relative mRNA expression of Tim4 in primary macrophages with silencing Tim4. Data represent the mean scores±SD. *P<0.05.

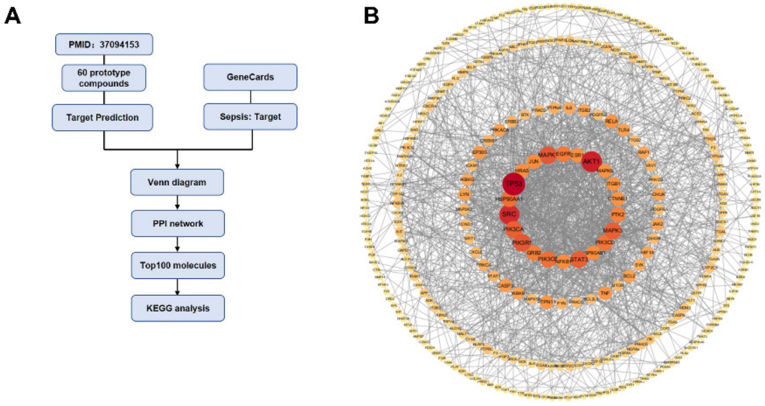

Fig. S8 (A) The workflow of network pharmacology. (B) PPI network of predicted targets for HSBD and sepsis visualized by Cytoscape.

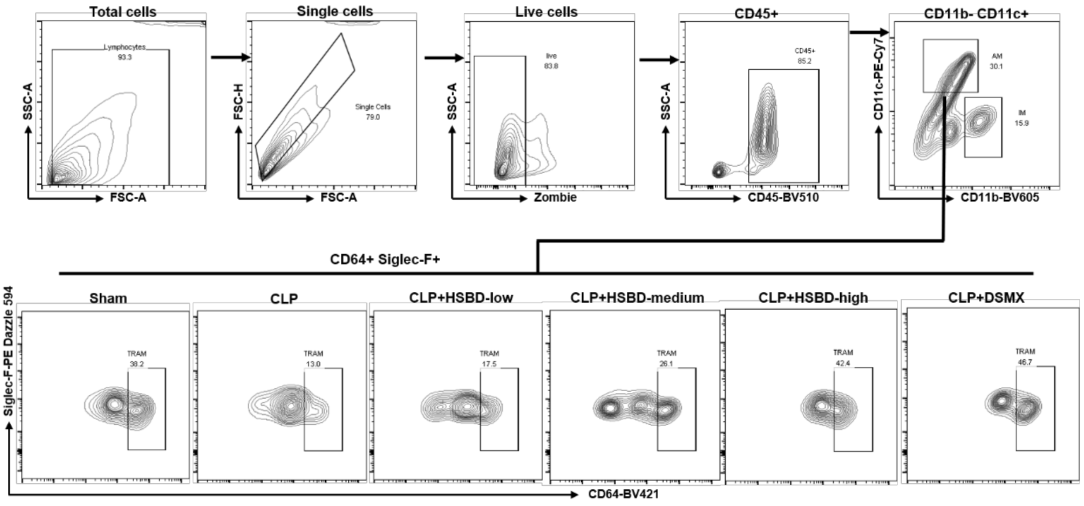

Fig. S9 The gating strategy of macrophages in BALF.

Table S1 Primer sequence of genes for used for the QPCR analysis.

Species	Gene	Forward primer (5'-3')	Reverse primer (5'-3')
Mouse	GAPDH	AGGTCGGTGTGAACGGATTTG	TGTAGACCATGTAGTTGAGGTCA
	Tim4	TTGATGATTCAAGGCCATCGTT	AGCTGGTGTCAGATAAAGCCA
	FoxO1	CCCAGGCCGGAGTTTAACC	GTTGCTCATAAAGTCGGTGCT
	ZO-1	GCCGCTAAGAGCACAGCAA	TCCCCACTCTGAAAATGAGGA
	Occludin	TTGAAAGTGCCACCTCCTTACAGA	CCGGATAAAAAGAGTACGCTGG
	TNF-α	GACGTGGAACTGGCAGAAGAG	TTGGTGGTTTGTGAGTGTGAG
	IL-6	TAGTCCTTCCTACCCCAATTTCC	TTGGTCCTTAGCCACTCCTTC
	iNOS	GTTCTCAGCCCAACAATACAAGA	GTGGACGGGTCGATGTCAC
	IL-10	GCTCTTACTGACTGGCATGAG	CGCAGCTCTAGGAGCATGTG
	IL-4	GGTCTCAACCCCCAGCTAGT	GCCGATGATCTCTCTCAAGTGAT
	Sin3a	CCAAGGAATTTGCGGCGTATC	GCAGACGCTTGCTTACACG
